# Multidimensional regulatory mechanisms of chicken immunity: focus on viral infection, intestinal microbiota and heat stress

**DOI:** 10.3389/fvets.2026.1837977

**Published:** 2026-06-03

**Authors:** Yanhong Wang, Min Yang, Jingying Zhao, Hao Wu, Kun Wang, Dawei Sun, Ru Zhang, Zhiqiang Xu, Zhenhui Cao, Changrong Ge, Junjing Jia, Lixian Liu, Zonghui Jian, Tengfei Dou, Xiannian Zi

**Affiliations:** 1 Faculty of Animal Science and Technology, Yunnan Agricultural University, Kunming, Yunnan, China; 2 Institute of Science and Technology, Chuxiong Normal University, Chuxiong, China; 3 Yunnan Academy of Animal Husbandry and Veterinary Sciences, Kunming, Yunnan, China; 4 College of Food Science and Technology, Yunnan Agricultural University, Kunming, Yunnan, China; 5 Faculty of Animal Husbandry and Veterinary Medicine, Yunnan Vocational and Technical College of Agriculture, Kunming, Yunnan, China

**Keywords:** avian-specific immunity, chicken, heat stress, immune regulation, intestinal microbiota, poultry disease control, viral immunosuppression

## Abstract

Chickens are a vital dietary protein source globally, yet their immune function is increasingly compromised by interconnected challenges including breed specific genetic heterogeneity, viral infections, intestinal microbial dysbiosis, and environmental stressors—all of which hinder sustainable poultry production. The chicken immune system, anchored by central immune organs (bursa of Fabricius, thymus) and peripheral immune organs (spleen, lymph nodes), relies on the coordinated interplay of innate and adaptive immunity to fend off exogenous pathogens. However, the holistic regulatory networks linking genetic background, pathogenic pressure, microbial communities, and environmental cues in shaping chicken immunity remain incompletely resolved. Immune related gene polymorphisms (e.g., MHC-B, TLR4) underpin interbreed differences in immune response magnitude and specificity, while viral pathogens such as Newcastle Disease Virus (NDV) and infectious bursal disease virus (IBDV) disrupt T/B lymphocyte dynamics, alter the expression of immune regulatory microRNAs (miR-155, miR-21) and pro−/anti-inflammatory cytokines (IL-1β, IFN-*γ*), and thereby perturb immune homeostasis. The intestinal microbiota, a key mediator of immune function, interacts with intestinal epithelial cells via metabolites (e.g., short-chain fatty acids, bile acids) or modulates the gut immune microenvironment through microbial transplantation, yet the specific targets of core functional taxa and their metabolites in chicken intestinal immunity along with comparative divergences from mammalian systems remain poorly defined. Environmental stressors, particularly heat stress, perturb amino acid metabolism, induce reactive oxygen species accumulation, and disrupt immune cell homeostasis, with synergistic immunosuppressive effects when combined with high density farming and nutritional deficiency. This review synthesizes chicken immune regulation by integrating genetic variation, viral pathogens, intestinal microbiota, and environmental stressors, and defines key regulatory pathways from a host-microbe-environment perspective.

## 1 Introduction

Domestic chickens are one of the world’s most widely consumed poultry and a critical source of high-quality dietary protein, with eggs also providing rich nutrition to underpin human food security and dietary structure (1–3). Flock health is the prerequisite for meeting consumer demands, and immune function acts as the core defense for chickens against pathogens and for sustaining normal growth and development. The chicken immune system comprises three fundamental components: immune organs (central and peripheral), immune cells (lymphocytes, mononuclear phagocytes, granulocytes, dendritic cells, etc.), and immune molecules (antibodies, cytokines, MHC, pattern recognition receptors (PRRs), and the complement system) (4–6). These components coordinate to form a complete system mediating immune defense, homeostasis, and surveillance, with immune regulation relying on the interplay of innate (physical barriers, cellular and molecular mediators) (7) and adaptive (humoral and cellular) (8) immune mechanisms. Pursuit of high poultry production yields is increasingly challenged by multiple stressors, including heat stress (9, 10), high stocking density (11, 12), and viral diseases (13), which cause severe economic losses to the poultry industry. Intensive farming exacerbates disease susceptibility due to genetic homogenization, immune deficiency, and live animal transportation (14), while the high prevalence of epidemics, limited pathogenic understanding, and overstocking in rural farms have led to antibiotic abuse, further compromising production management and increasing economic losses.

**Figure 1:**
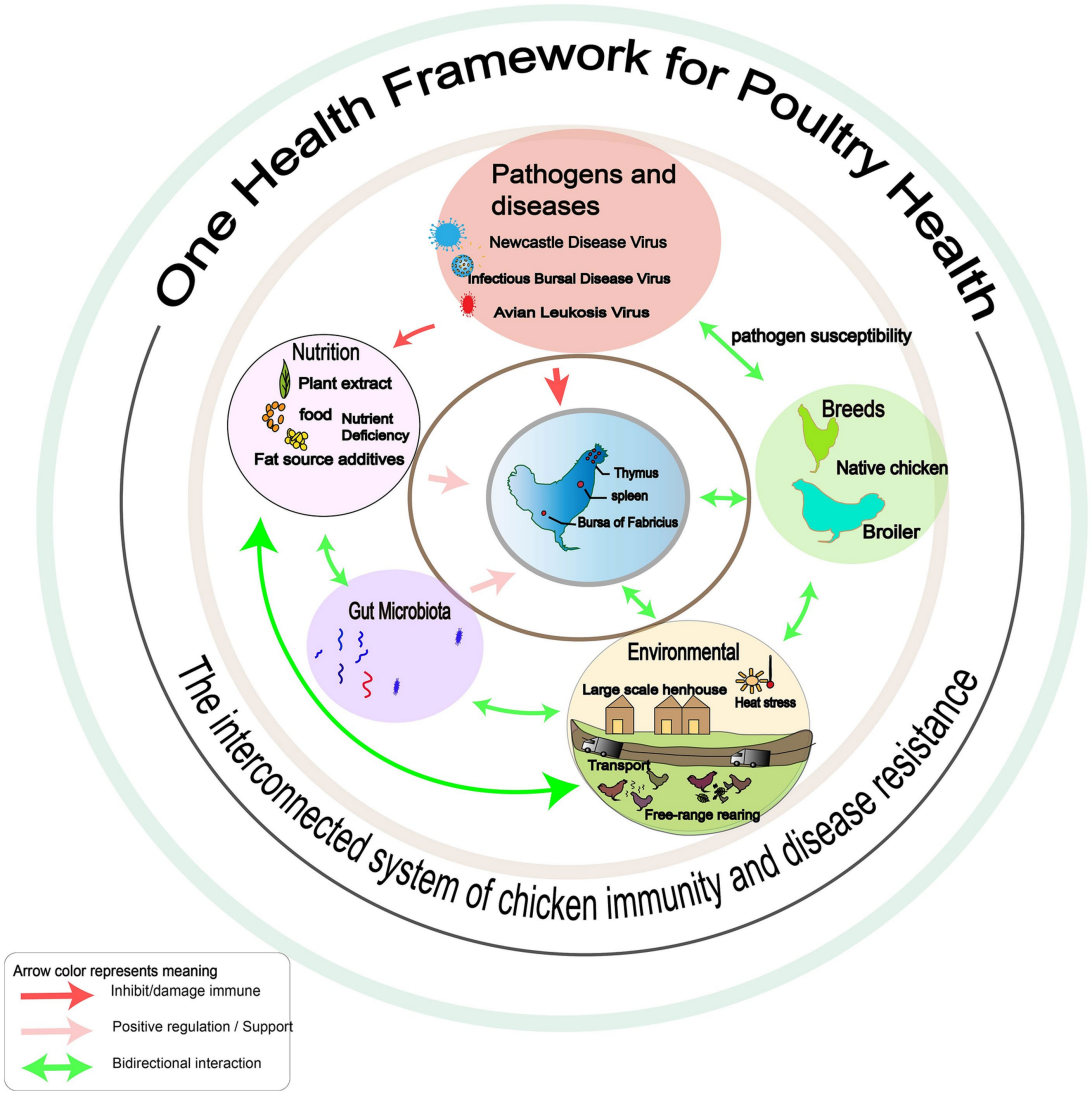
Conceptual framework of the One Health concept applied to chicken immunity. Central immune organs (thymus, spleen, bursa of Fabricius) are modulated by five key external factors. Interactions include: (1) Pathogen susceptibility to Newcastle Disease Virus, Infectious Bursal Disease Virus, and Avian Leukosis Virus; (2) Breed-specific responses (native vs. broiler); (3) Environmental perturbations (heat stress, farming practices); (4) Modulation by gut microbiota; and (5) Nutritional influence (feed, additives, deficiency). Arrows denote interaction types: red (inhibition/damage), pink (positive support), green (bidirectional interaction).

Despite advances in the field, chicken immunity research still faces multiple unresolved core challenges, with research directions remaining fragmented and studies on the crosstalk between regulatory factors severely lacking. Most existing work focuses on single-factor regulation, including breed (15–17), viral pathogens including IBDV (18), *Salmonella Typhimurium* (19), Avian Influenza Virus (AIV) (20), NDV (21), Avian Leukosis Virus (ALV) (22), intestinal microbiota (23, 24), and environmental stressors including free-range rearing (25), heat stress (26), transportation (27), nutritional deficiency (28) (Figure 1). By contrast, investigations into the synergistic effects of breed genetics and viral infection, intestinal microbiota and environmental stress, and the tripartite interaction of virus-microbiota-host immunity are inadequate, and the molecular network underlying the multi-factor coordinated regulation of chicken immunity remains unelucidated. With the modernization of animal husbandry, chicken rearing environments are additionally threatened by environmental pollutants, and avian immunity research lacks a unified integrated framework—such as a genetic-environment-microbiota interaction model for chicken immune regulation and a balance model for evolutionary conservation and species specificity of avian immune signaling pathways. Moreover, the molecular trade-off between enhanced disease resistance and improved growth performance (29) remains a long-standing core conundrum in the field. Avian-specific immune regulatory mechanisms are also poorly characterized: Most studies rely on extrapolations from mammalian immunity, potentially overlooking chicken-specific regulatory rules, research on chicken-unique immune organs, molecules, and pathways [bursa of Fabricius (30), NF-κB1 (31), bursa-dependent B-cell maturation (32)] is still limited. Compared with mammals, the specific targets of core functional gut microbes and their metabolites in chicken intestinal immunity remain unclear. Additionally, current methodologies remain limited: most studies rely on *in vitro* cell lines or acute stress models that do not reflect the chronic, multi-factor conditions of commercial farms. As a result, findings often fail to translate into practical interventions.

Therefore, the immunity–microbiome–environment axis is proposed as an integrated conceptual framework comprising three core modules: host immunity, intestinal microbiome, and external environmental stressors. Environmental factors such as heat stress and pollutants remodel the composition of intestinal microbiota, which in turn regulates intestinal barrier function and immune homeostasis. Viral infection further serves as a key internal trigger that synergistically exacerbates immune disorders under combined environmental and microbial perturbation. Unlike conventional single-factor research paradigms, this framework emphasizes dynamic crosstalk and synergistic interactions among these multi-layered determinants, providing a holistic theoretical reference for systematically elucidating the immune regulatory mechanisms in chickens. Genetic background, viral infection, intestinal microbiota and environmental stressor interact closely rather than acting independently. They share overlapping signaling pathways to jointly regulate chicken immune homeostasis, which is the core basis for constructing the immunity-microbiome-environment axis. This review systematically constructs a multidimensional interactive regulatory network of chicken immunity by integrating genetic, viral, microbial and environmental factors, clarifies the species-specific immune mechanisms of chickens (e.g., bursa of Fabricius function, TLR family contraction evolution), and proposes a novel immunity-microbiota-environment axis conceptual framework. Our findings provide a theoretical basis for green and sustainable poultry production, and also offer comparative biological references for human intestinal immunity and antiviral research. This framework aims to guide future research toward integrated intervention strategies and to support the development of sustainable poultry production systems.

## 2 Chicken immune system

### 2.1 Immune organs

The developmental degree of immune organs can, to a certain extent, reflect the developmental level of the body’s immune system, and this developmental status in turn impacts the body’s immunity. Central immune organs (thymus, bursa of Fabricius) primarily function in the production and maturation of immune cells. Thymocytes mainly originate from immature T lymphocytes, while thymic stromal cells primarily derive from thymic epithelial cells (33). Thymic epithelial cells are the core components of the thymic microenvironment and are classified into cortical thymic epithelial cells and medullary thymic epithelial cells (mTECs) (34). cortical thymic epithelial cells mediate T-cell positive selection through a unique antigen processing system, while mTECs restrict the ectopic expression of antigens in peripheral tissues and cooperate with dendritic cells to maintain T-cell tolerance (35). Among these processes, single-positive T cells that have undergone positive selection migrate to the corticomedullary junction and medullary region, where mTECs and thymic dendritic cells (DCs) present self-antigens (especially tissue-restricted antigens, TRAs) on the surface of MHC molecules (36, 37). T cells with high affinity for self-pMHC complexes undergo apoptosis and are eliminated, while some T cells with intermediate affinity differentiate into Treg. T cells with no affinity for self-pMHC fail to bind and are cleared by “death by neglect” due to insufficient survival signals. Low-affinity T cells bind weakly to self-pMHC, acquire survival signals through positive selection, migrate to the periphery, and gain MHC restriction (38). T cell negative selection is a core process in the establishment of central immune tolerance, occurring in the thymic medulla (39). Its primary function is to eliminate T cells with high affinity for autoantigens, thereby preventing autoimmune diseases. After the cortical stage, thymocytes migrate to the medulla, where mTECs recruit positively selected thymocytes and provide environmental signals. Through negative selection, self-reactive cells are cleared, and Foxp3^+^CD4^+^ Tregs are generated via sublineage differentiation to mediate self-tolerance (40, 41). Three core regulatory factors govern the establishment of the thymic central immune microenvironment, normal T-cell development, and central immune tolerance: *Foxn1*, *Aire*, and *Fezf2*. *Foxn1* is the sole core transcription factor for the fate specification, maturation, and functional maintenance of thymic epithelial cells, its absence directly leads to thymic developmental arrest (42). *Aire* and *Fezf2* are two core transcription factors in mTECs that mediate T-cell negative selection (43). *Aire* regulates the expression of approximately several thousand TRAs, including various tissue-specific antigens, and is mainly expressed in mature mTECs, activated via the NF-κB signaling pathway. However, some antigens require cross-presentation by DCs to effectively induce negative selection (44, 45). *Fezf2* functions independently of *Aire*: it is primarily expressed in mTECs (46, 47), directly binds DNA sequences to activate TRA expression through an Aire-independent pathway, and may be involved in chromatin opening and the assembly of transcription initiation complexes (48). Together, *Aire* and *Fezf2* regulate the comprehensive expression of TRAs in mTECs, ensuring the thoroughness of T-cell negative selection and serving as a key barrier against autoimmunity. These three core regulatory factors form a complete regulatory cascade for TEC differentiation and maturation, antigen presentation, and clearance of self-reactive T cells—critical for the body to avoid autoimmune diseases. Through synergistic interactions, these thymic immune cells respond to foreign antigens and further regulate the adaptive immune system (49).

As oviparous animals, chicken embryos cannot acquire maternal immune cells via the placenta, unlike viviparous animals. In addition to the thymus (a key central immune organ), the bursa of Fabricius—a specialized gut-associated lymphoid tissue organ—relies on a B-cell development pathway unique to avians. This specialization compensates for the gap in immune function during embryonic and early postnatal stages caused by oviparous development. The bursa of Fabricius acts as a secondary immune organ involved in immune responses (50) and facilitates the rearrangement of surface antibodies on B lymphocyte precursors, as well as the binding of B lymphocyte surface receptor complexes to intestinal antigens (51). Formed in the cloaca (the junction between the intestine and the external environment), the bursa of Fabricius enables early exposure to intestinal microbiota antigens. Dynamic changes in the microbiota can influence the maturation of bursal B cells (52), offsetting insufficient antigen exposure in oviparous animals before birth. While traditional views hold that the bursa of Fabricius loses function in adult chickens, recent single-cell data indicate it retains immune regulatory capacity. Beyond B cells, the bursa contains abundant non-B cell populations, including epithelial cells, dendritic cells, macrophages, and fibroblasts. Following infection, it regulates B-cell subset conversion (decreased IgM^+^ B cells, increased IgA^+^ B cells) via intercellular communication networks to maintain local immune homeostasis (53). Currently, no studies have directly demonstrated that bursal peptides (bursins) secreted by the bursa of Fabricius directly regulate the growth or metabolism of intestinal bacteria through mechanisms such as receptor binding, as *in vivo* validation is lacking—this represents a key direction for future research. The B-cell maturation pattern of the bursa of Fabricius differs from the immune strategies of viviparous mammals. Whether this implies the bursa of Fabricius is not only a B-cell maturation organ but also a core hub of avian immunity, and whether premature involution of the bursa of Fabricius induced by intensive farming is a key cause of immune deficiency and frequent diseases, remains undetermined.

Peripheral immune organs, including the spleen and gut-associated lymphoid tissues (GALT), are responsible for the activation of immune cells. The anatomical compartments of the chicken spleen, namely ellipsoids, periellipsoidal lymphoid sheaths, and diffuse lymphoid tissues, comprise diverse immune cells such as B cells, T cells, and macrophages. Upon infection or host injury, numerous PRRs expressed on myeloid cells recognize antigens, and the selective expression of PRRs in distinct cell types within specific regions of the spleen shapes the characteristics of early innate immune responses and subsequent adaptive immune responses (54, 55). The chicken spleen not only functions in hematopoiesis and erythrocyte clearance but also mediates a broad spectrum of immune activities (56), with its core roles involving the recognition and elimination of blood-borne antigens as well as the initiation of adaptive immunity (57). Given the absence of a typical marginal zone structure in the chicken spleen, this immune function is executed by ellipsoids and peri-ellipsoidal lymphoid sheaths. The efficient capture and presentation of antigens rely on the coordinated recognition of pattern recognition receptors including Toll-like receptors, which provides a pivotal signal for the initiation of adaptive immune responses. Upon the entry of blood-borne pathogenic antigens into the peri-ellipsoidal lymphoid sheaths of the chicken spleen, Toll-like receptors on ellipsoid-associated cells first recognize pathogen-associated molecular patterns (58, 59), triggering intracellular signaling cascades such as the NF-κB pathway, thereby activating ellipsoid-associated cells and upregulating the expression of phagocytosis-related receptors. In addition to immune functions, the spleen serves as a key component of the neuro-immune axis, where dense sympathetic nerve fibers innervate the spleen and precisely regulate immune responses via catecholaminergic signals, predominantly norepinephrine (60). Intestinal dendritic cells contribute to the establishment of immune tolerance to commensal flora and food antigens in the gut, maintaining intestinal immune homeostasis, which represents the core molecular mechanism underlying the development of intestinal immune tolerance (61). The immune regulatory mechanism of GALT is predominantly executed via a three-step cascade: recognition, activation, and effector function. Specifically, DCs and macrophages in GALT recognize structural features of polysaccharides from commensal microbes (e.g., *β*-1,3-glucan and mannan) through pattern recognition receptors such as Dectin-1 (62, 63). This recognition exhibits remarkable microbial specificity, and polysaccharides derived from different bacterial genera (e.g., Lactobacillus exopolysaccharides, Bacteroidetes polysaccharides) differ in their binding affinity to PRRs (64, 65). Upon recognition, immune cells become activated. Ligation between polysaccharides and PRRs activates the MAPK and NF-κB signaling pathways, promoting DC maturation (66), enhancing their antigen-presenting capacity, and subsequently directing the differentiation of T cells toward Treg subsets, while facilitating B-cell secretion of secretory immunoglobulin A (67). Finally, mucosal defense is reinforced by enhancing the tight junction function of intestinal epithelial cells via the upregulation of ZO-1 and occludin expression. Mucosal immunity and physical barrier function act in synergy to maintain intestinal homeostasis (68). Notably, the structure of chicken GALT (e.g., the specialized distribution of the cecal tonsil) displays distinct species-specific characteristics compared with those of mammals. The mechanism underlying the activation of γδ T cells, the dominant T-cell subset in chicken GALT, by microbial polysaccharides remains incompletely understood, representing a key issue warranting further investigation in avian immune research. Both chickens and mammals adopt a central–peripheral two-tier immune system; the key difference is that the mammalian bone marrow serves both hematopoietic and B-cell maturation functions (69), the chicken bone marrow is solely responsible for hematopoiesis, and B-cell maturation depends on the unique bursa of Fabricius. Mammalian antibody classes include IgM, IgG, IgA, IgE, and IgD (70), chickens possess only the first three classes.

### 2.2 Immune cells

Chickens are susceptible to various diseases, and in-depth research on the composition and immune functions of chicken immune cells is crucial for preventing avian diseases and promoting the healthy, sustainable development of the poultry industry. Chicken immune cells mainly include lymphocytes (e.g., T cells mediating cellular immunity; B cells and natural killer (NK) cells involved in humoral immunity), the mononuclear phagocyte system (including monocytes and macrophages with phagocytic and antigen-presenting functions), granulocytes (neutrophils acting in bacterial and fungal infections, eosinophils regulating parasitic infections and allergic reactions, basophils releasing bioactive substances during allergic responses), and DCs playing a key role in adaptive immunity. Lymphocytes primarily consist of T cells (cellular immunity), B cells, and NK cells (humoral immunity). Both T and B cells originate from bone marrow hematopoietic stem cells: in poultry, B cells differentiate in the bursa of Fabricius, while T cells mature in the thymus. Avian CD8^+^ T cells, upon antigen stimulation, differentiate into cytotoxic T cells and Treg. Cytotoxic T cells are critical for controlling avian infectious bronchitis virus in poultry and can provide long-term protection through memory T cells (71). While Cytotoxic T cells specifically recognize and eliminate tumor or virus-infected cells, Treg cells secrete immunosuppressive cytokines such as IL-10 and TGF-*β* to restrain excessive immune responses and maintain self-tolerance (72). Chicken B-cell precursors originate from yolk sac hematopoietic stem cells. After migrating to the embryonic spleen, they simultaneously complete V(D) J gene rearrangement of both heavy and light chains; rearranged B-cell precursors then migrate to the bursa of Fabricius for colonization and initial functional activation— a prerequisite for B-cell functional maturation (73). B-cell activating factor is critical for B-cell survival and maturation in the bursal microenvironment (74). B-cell antigen presentation can occur via antigen-specific (B-cell receptor (BCR)-dependent) or antigen-non-specific (BCR-independent) mechanisms. The core function of BCR is to specifically bind antigens (75), and Bu-1 is a specific marker of chicken B cells. Increased numbers of Bu-1^+^ B cells suggest that IL-4 may be involved in B-cell proliferation or activation (76, 77), cooperating with Tc cells to clear pathogens (78). NK cells are key components of the innate immune system that interact with other immune cells, secrete cytokines, and play important roles in regulating adaptive immune responses. Initially thought to differ from cytotoxic T cells, NK cells lack antigen-specific TCRs and are not MHC-restricted. Th1-mediated factors such as type 1 cytokines (e.g., IFN-*γ*) can enhance NK cell proliferation and cytotoxicity (79). Additionally, intestinal intraepithelial NK cells are important for innate immune responses, responding rapidly to viral infections and eliminating infected or transformed target cells through cytotoxicity (80).

The mononuclear phagocyte system comprises monocytes and macrophages with phagocytic and antigen-presenting functions. Monocytes are generated in the bone marrow and released into the bloodstream, representing the largest cells among leukocytes (81, 82). Macrophages initiate and regulate host immune responses, and their abundance in the intestinal mucosa makes them critical for resisting microbial invasion (83). IFN-*γ* can activate macrophages, upregulate MHC-I and MHC-II molecule expression, block viral replication, and eliminate intracellular pathogens (84). Although chickens are thought to exhibit Treg/Th2-like responses, the lack of clear evidence for typical transcription factors (e.g., *GATA3*) has led to controversy regarding the adaptive immune polarization pattern in poultry. Additionally, chicken blood monocytes induced by granulocyte/macrophage colony-stimulating factor and stimulated with lipopolysaccharide (LPS) after 3 days of culture upregulated pro-inflammatory cytokine expression, phagocytosed bacteria, and produced nitric oxide—conditions most favorable for obtaining pro-inflammatory M1-like chicken macrophages (85).

Chicken granulocytes include neutrophils (bacterial/fungal infections), eosinophils (parasitic infections/allergic reaction regulation), and basophils (bioactive substance release during allergic responses). Morphological and physiological characteristics of avian peripheral blood leukocytes have been analyzed in accordance with international standards. High-resolution color micrographs of early postnatal chicken samples revealed eosinophils with typical bilobed “eosinophilic” nuclei, eccentric multilobed nuclei, and rod-shaped nuclei with distinct nuclear contours. Basophils are the smallest granulocytes, with slightly basophilic cytoplasm containing dense basophilic granules of varying sizes—smaller than those of other granulocytes (86). Eosinophils are short-lived cells continuously generated by bone marrow hematopoietic stem cell differentiation. Upon parasitic infection or inflammatory stimulation, eosinophils rapidly accumulate in target tissues. Fully activated eosinophils release extracellular traps composed of mitochondrial DNA and tissue-destructive granular proteins to capture and kill bacteria, similar to neutrophils—though this cytotoxic response only occurs under inflammatory conditions and requires strong stimulation by cytokines such as IFN-*γ* and high levels of IL-5 to induce DNA network formation (87). For example, after *Salmonella Typhimurium* infection, eosinophil numbers increase in the follicular cortex, follicular spaces, and within/around blood vessels of the chicken bursa (88). Additionally, neutrophil counts and functions (oxidative burst, chemotaxis) did not respond to stress, but increased in stressed hens (89). Hens reared in alternative systems exhibited lower relative neutrophil numbers and higher total leukocyte counts (90). One-day-old specific pathogen-free (SPF) chicks infected with ALV-J showed macrocytic hypochromic anemia, leukocytosis, heterophilia, and thrombocytosis in blood routine tests conducted at 1, 2,3, 4, and 5 months post-infection (91).

DCs differentiate from bone marrow cells and are widely distributed in various tissues. They recognize, uptake, and process exogenous antigens, bind antigenic peptides to MHC molecules for surface presentation, activate T and B cells, and initiate cellular and humoral immune responses (92). DC maturation and function are regulated by microorganisms and the environment, enabling them to modulate immunity against pathogens. Immature DCs are highly efficient at antigen capture; antigen processing promotes MHC molecule migration, upregulates co-stimulatory molecule expression, induces cytokine production, and remodels cell morphology—triggering maturation and initiating immune responses (93, 94). DCs possess unique immune functions, playing key roles in inducing innate immunity and activating adaptive immunity. They can also act as immune regulators and vaccine adjuvants to enhance cellular immunity, contributing to modern vaccination strategies (95). Under homeostatic conditions, immature DCs in peripheral tissues exhibit strong endocytic and phagocytic capabilities, monitoring microorganisms via PRRs such as toll-like receptors. The contraction of the TLR family and specialization of the PRR recognition spectrum in poultry represent immune reduction strategies adapted to flight-related metabolic constraints, though their molecular mechanisms remain unclear (96, 97). Upon PRR activation by pathogen- or tissue damage-associated “danger signals,” DCs mature—undergoing morphological changes, reducing antigen uptake/processing capacity, upregulating MHC and co-stimulatory molecule expression, and secreting cytokines to regulate immunity. Additionally, the transforming growth factor *β* receptor signaling pathway is critical for the development of intestinal-specific DCs, through which DCs maintain intestinal immune homeostasis (98). NF-κB is a nuclear factor involved in immune cell activation and inflammatory signaling pathways (99). Present antigenic peptides to CD4^+^ T cells via MHC-II molecules (100), stimulates NO synthesis in the chicken HD11 macrophage cell line, and initiates macrophage responses to microbial agonists (e.g., LPS, peptidoglycan) (101)—forming a “DC-T cell-macrophage” positive amplification loop. NO is a key effector molecule for macrophage bactericidal activity, directly disrupting the metabolism and membrane structure of intracellular pathogens such as *Salmonella*. Both chickens and mammals possess conserved core subsets of innate immunity (macrophages, dendritic cells, NK cells) and adaptive immunity (T and B cells).

Recent advances in single-cell RNA sequencing (scRNA-seq) have enabled high-resolution mapping of immune cell heterogeneity, differentiation trajectories, and functional states in chickens during homeostasis, infection, and vaccination. These studies have uncovered previously unrecognized cell subsets, dynamic gene expression changes, and cell–cell communication networks that are masked in bulk analyses. As summarized in Table 1, scRNA-seq studies have revealed, for instance, that IBDV infection shifts bursal B-cell populations from IgM^+^ to IgA^+^, and that ALV-J infection alters T-cell subset proportions through pro-apoptotic factors. Such findings provide a detailed perspective on immune cell lineage differentiation and functional plasticity in chickens. However, gaps remain in the understanding of subset functions and regulatory mechanisms: *in vitro* studies rely on limited cell lines (e.g., HD11 macrophages, DF-1 fibroblasts), and the lack of long-term primary immune cell culture systems hinders simulation of *in vivo* cellular functions in complex microenvironments. Immune cell composition and function vary significantly among chicken breeds (e.g., Wenchang chickens, broilers, layers), and the mechanisms by which MHC molecular polymorphisms influence immune responses remain unclear—resulting in a lack of breed-specific immune prevention and control technologies. These issues are under active investigation. Future research should focus on the antigen recognition mechanisms of γδT cell subsets, their unique pathways in anti-tumor (e.g., Marek’s disease virus-induced tumors) and anti-inflammatory responses, and the regulatory effects of intestinal microbiota metabolites [e.g., short-chain fatty acids (SCFAs)] and natural active substances (e.g., purple corn anthocyanins, plant polysaccharides) on immune cells.

**Table 1:** Relevant applications of single-cell genomics in chicken immunity.

Variety	Processing type	Cell count/type	Key findings	Cite
SPF chicken	Infectious Bursal Disease Virus	Follicular cells	Infection decreases IgM^+^ B cells and increases IgA^+^ B cells in bursal follicles, with viral particles accumulating in bursal epithelial (basal) cells.	(224)
SPF chicken	Avian Leukosis Virus - Subgroup J	T cell population, PBMCs	In ALV-J-infected chicken PBMCs, T cell clusters 6/7 show reduced proportions due to ITPR2 (pro-apoptotic factor) upregulation. At 21 dpi, immune gene *Irf7* is upregulated in clusters 0–3, while anti-apoptotic gene BCL2L10 is upregulated in clusters 0–1, 3–5.	(225)
SPF chicken	Influenza A (H5N1) Virus	16 cell types (19 cell clusters) in lung tissue	At the single-cell level, it has been confirmed that AECII is the primary target cell for H5N1 infection, and its apoptosis is the initiating factor for lung injury.	(226)
SPF chicken	Infectious Bronchitis Virus	Cells in the kidney, bursa of Fabricius, and trachea tissues	Infection causes upregulation of IL18 and IL1B transcription in renal macrophages and increased STAT gene expression in distal tubule and collecting duct cells.	(53)
Dekalb White Leghorn laying hens	–	Peripheral blood leukocytes	B cell cluster 10 shows high expression of BCR genes, MHC II, IGLL1, CXCR4, BAFF/BAFF-R, HMGB1, and ribosomal proteins.	(227)
Local Chinese yellow-feathered breed	Eimeria tenella	7,394 cells	APOB-positive intestinal epithelial cells decrease, and proliferative T cells increase.	(228)
White Leghorn chickens with resistance 63, susceptible 72, and their F1 hybrids	Marek’s Disease Virus	Spleen immune cells	ICP4/MEQ are widely expressed in infected cells, with parental allele bias in HDAC1/8 (F1 immune cells) and SNORA68/72 (T cell subsets) during infection.	(229)
Beijing-You chickens and Kobao broiler chicken	*S. typhimurium*	Spleen CD45 + immune cells	Discovery of inflammatory regulatory signaling pathways between macrophages and T cells	(230)

Summary of published single-cell RNA-sequencing studies on chicken immune responses to pathogenic and physiological challenges. PBMCs, peripheral blood mononuclear cells; AECII, alveolar epithelial type II cells; IBD, inflammatory bowel disease; dpi, days post-infection.

### 2.3 Synergistic mechanisms of innate and adaptive immunity in chickens

The chicken immune response is achieved through the synergy of innate and adaptive immunity, forming a highly integrated functional network via PRR recognition, NF-κB signal activation, DC antigen presentation, T/B cell differentiation, and regulation of the neuro-endocrine-immune network. The immune response is divided into three stages: pathogen recognition and signal initiation, immune cell activation and regulation, and effector clearance with immune memory formation. The chicken innate immune system plays a pivotal role in activation and sensitization. As the first line of defense, it is anchored by sentinel cells (macrophages, DCs, NK cells) and PRRs expressed on epithelial barriers—including the TLR and RLR families—with distinct avian-specific characteristics. The TLR family undergoes “contractile evolution,” comprising only 10 functional gene members (*Tlr1a*, *Tlr1b*, *Tlr2a*, *Tlr2b*, *Tlr3*, *Tlr4*, *Tlr5*, *Tlr7*, *Tlr15*, *Tlr21*)—significantly fewer than the 13 found in mammals (102)—yet its recognition spectrum exhibits specialized adaptability. For example, TLR3/TLR7 form a specific recognition network for avian RNA viruses (103, 104). During rapid inflammatory responses, the immune system acts as a sensory system, receiving signals and transmitting them to the brain via humoral pathways (e.g., the cytokine IL-1 stimulates nerve cells to uptake glucose, supporting central nervous system metabolism) or neural pathways to trigger physiological reactions (105). Recognition of pathogen-associated molecular patterns or damage-associated molecular patterns by PRRs rapidly activates downstream signaling pathways (e.g., NF-κB), promoting immune cells to secrete pro-inflammatory cytokines and chemokines, enhancing phagocytic and bactericidal capacities of phagocytes, and quickly initiating inflammatory responses to resist pathogen invasion (106, 107). When pathogen-associated molecular patterns bind to toll-like receptors, they trigger an initial alarm, initiate intracellular signal transduction, and induce massive pro-inflammatory cytokine production, which further propagates inflammatory signals in DCs and intraepithelial lymphocytes (108). Upon activation, TLR4 initiates intracellular signaling cascades that activate core transcription factors including NF-κB, activator protein 1, signal transducers and activators of transcription, and interferon regulatory factors, thereby orchestrating the overall inflammatory response (109).

Adaptive immunity exhibits specificity and memory, with T/B lymphocytes as the core regulators. It relies on the cascade signal “innate immune sensitization to adaptive immune activation” to achieve precise targeting and long-term protection against pathogens, encompassing humoral immunity, cellular immunity, and neuro-endocrine-immune network regulation. Adaptive immunity responds to specific pathogens more precisely and efficiently, synergizing with the innate immune system to construct a robust immune defense for chickens. Adaptive immune recognition involves the body recognizing MHC-presented antigenic peptides via TCRs (110) or BCRs directly recognizing antigens to initiate specific immune responses. Characterized by high specificity, memory, and tolerance, it plays a key role in immune defense (111). Humoral and cellular immunity consist of lymphocytes and effectors (mainly immunoglobulins and MHC molecules), which sense and eliminate pathogens. Through the diversity of T/B cell receptors, adaptive immunity accurately identifies antigenic variations, compensating for the limitations of innate immunity and representing an inevitable evolutionary advancement of immune function (112). The high polymorphism of the chicken MHC-B gene is the core driver of inter-individual differences in disease resistance. However, intensive selection for production traits may reduce MHC-B diversity in commercial chicken lines, potentially contributing to increased disease susceptibility. Further validation through population genetics studies is required. Genetic resistance in local chicken breeds is often attributed to MHC-B polymorphisms; yet, genome-wide association studies suggest resistance may be jointly regulated by multiple genes rather than a single gene effect. While associations between MHC-B polymorphisms and disease resistance have been widely reported (113, 114), the specific antigen presentation mechanisms remain unclear and require further verification using structural biology and single-cell technologies. Both chickens and mammals exhibit highly conserved immune response cascades, following the pattern of innate immunity initiation, adaptive immunity amplification, and immune memory maintenance, which rely on PRR recognition, T–B cell cooperation, as well as immune tolerance and feedback regulation.

**Figure 2:**
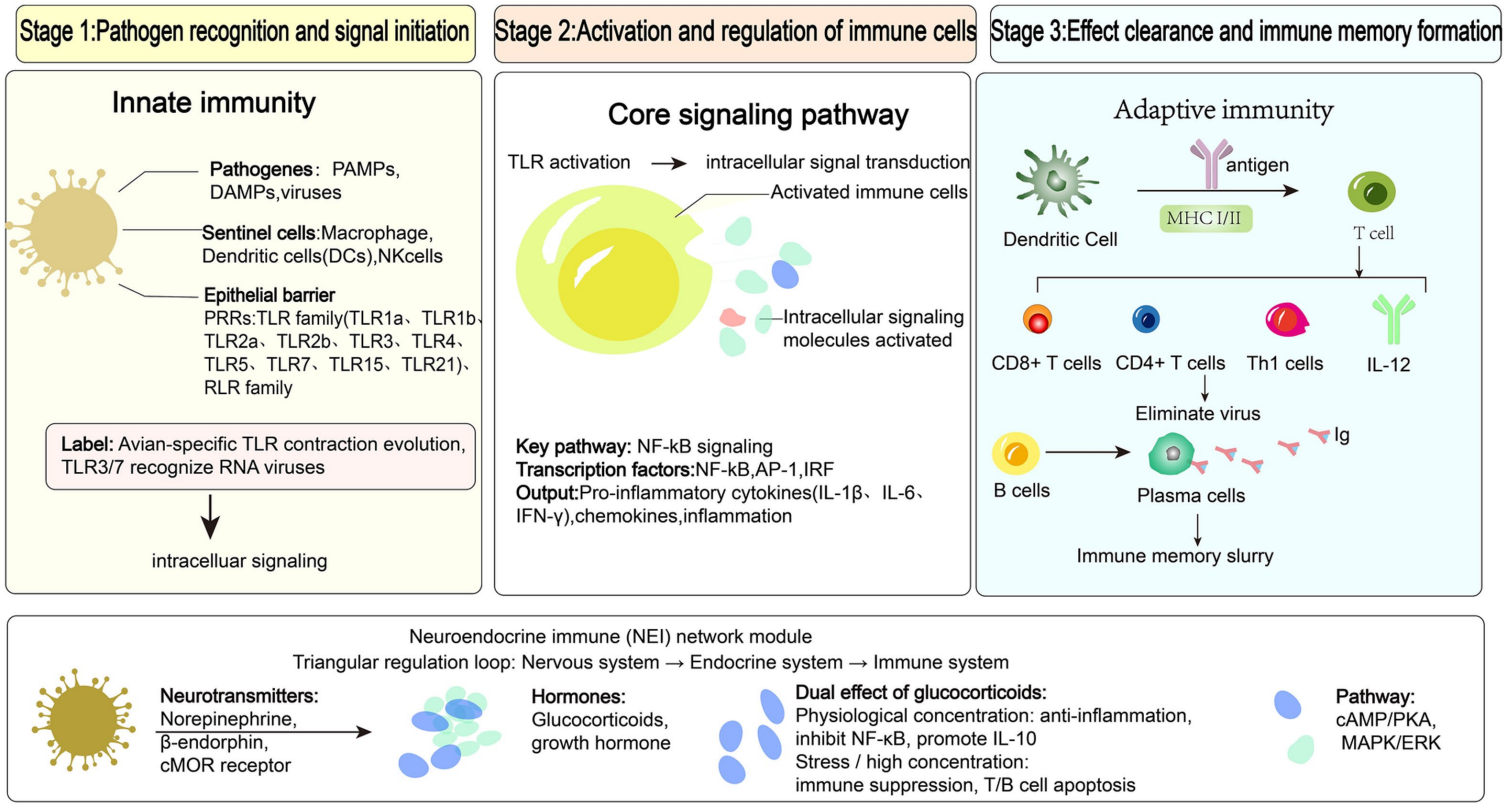
Schematic of the chicken antiviral immune response and neuroendocrine-immune (NEI) regulatory network. This model depicts three sequential immune stages and an integrated NEI module: Stage 1 (Innate immunity): Pathogens are detected by sentinel cells (macrophages, DCs, NK cells) and epithelial PRRs (TLR/RLR families), with avian-specific TLR3/7 recognizing RNA viruses to initiate intracellular signaling. Stage 2 (Core signaling): TLR activation drives NF-κB–mediated transcription of pro-inflammatory cytokines (IL-1β, IL-6, IFN-*γ*) and chemokines, activating immune cells. Stage 3 (Adaptive immunity): Dendritic cells present antigens to T cells via MHC I/II, promoting CD8^+^/CD4^+^ T cell differentiation and B cell–derived plasma cells to clear viruses and form immune memory. NEI module: A nervous–endocrine–immune triangular loop modulates immunity; glucocorticoids exert concentration-dependent dual effects (anti-inflammatory at physiological levels, immunosuppressive under stress) via cAMP/PKA and MAPK/ERK pathways.

Meanwhile, the neuro-endocrine-immune network is a complex regulatory system with feedback mechanisms among the nervous, endocrine, and immune systems (Figure 2). The neuroendocrine system regulates immune function by releasing neurotransmitters (e.g., norepinephrine), neuropeptides (e.g., endorphins), and hormones (e.g., glucocorticoids, growth hormones), which act on adrenergic receptors, cholinergic receptors, steroid receptors, and growth hormone receptors on the surface of immune cells (e.g., T/B cells, macrophages). For example, neuropeptides exert immune-enhancing effects: chickens express six endogenous opioid peptides, including *β*-endorphins (the main active form of endorphins), which can activate the chicken *μ*-opioid receptor. Chicken μ-opioid receptor transmits signals by inhibiting the cAMP/PKA pathway and activating the MAPK/ERK pathway (115), participating in immune processes. Stress hormones exhibit bidirectional regulation: synthetic glucocorticoids at physiological concentrations significantly reduce the mRNA expression of NF-κB1 (a p65 homologous gene) in chicken splenocytes, simultaneously inhibiting the secretion of pro-inflammatory cytokines (e.g., IL-1β, IL-6) and increasing the level of the anti-inflammatory factor IL-10 (116). Under extreme conditions (e.g., heat stress, high stocking density), excessive glucocorticoids can induce T/B cell apoptosis, leading to immunosuppression—a mechanism particularly prominent in intensive chicken flocks and an important cause of frequent diseases (117, 118). While these signaling pathways are well-characterized in mammals, functional evidence in chickens remains limited. For instance, the role of the cAMP/PKA pathway in chicken *μ*-opioid receptor signaling has been confirmed (115), but the full NEI network in avian species requires further investigation. This interaction is mediated by chemical signaling molecules (e.g., neurotransmitters/neuropeptides, hormones, cytokines) and shared receptors among the nervous, endocrine, and immune systems. Unfortunately, most existing studies rely on “extrapolating chicken mechanisms from mammalian models,” with limited analysis of chicken-specific molecular regulatory nodes. Additionally, current research focuses on the impact of single stressors (e.g., acute heat stress, LPS-induced immune stress) on the chicken immune network. However, in intensive farming, chicken flocks face superimposed multiple stressors (heat stress, high density, feed stress, and subclinical pathogen infection). The synergistic regulatory rules of the immune network and key thresholds (e.g., the critical glucocorticoid concentration inducing immunosuppression) remain unclear. Furthermore, long-term tracking studies on the remodeling effects of acute/chronic/repeated stress on the immune network are lacking, creating a gap between laboratory conclusions and practical application in poultry production. Understanding these synergistic mechanisms provides the foundation for analyzing how genetic variation and viral infections perturb chicken immunity, which will be discussed in the following section.

## 3 The influence of genetics and viral infections on chicken immunity

### 3.1 The influence of genetics on chicken immunity

The immune function of chickens is the result of the combined effect of genetic factors and the environment. Genetics is the core intrinsic basis of immune differences, directly influencing chickens’ susceptibility to pathogens, the intensity of immune responses, and disease resistance. Populations with richer genetic diversity and larger effective population sizes tend to have stronger evolutionary capabilities—i.e., more prominent adaptive potential—when responding to ecological pressure (119). Chicken flocks with high genetic diversity have more diverse immune responses, which can reduce the risk of large-scale disease outbreaks. In contrast, intensive selection for production traits in commercial lines reduces genetic diversity at immune-related loci, a phenomenon sometimes accompanied by increased inbreeding and susceptibility to immune deficiencies. For instance, artificial selection of chickens can lead to a reduction in the variability of genes encoding immune system components, which may weaken the resistance of poultry to certain diseases (120). The underlying mechanisms of immunoglobulin diversity, including junctional diversity, somatic hypermutation, and gene conversion, are conserved across Hy-Line Brown chickens, Lueyang black-bone chickens, and Beijing-You chickens, compensating for the deficiency of V(D)J recombination (121). Genomic mapping of a total of 72 chicken genetic variation populations—including 9 Iranian native ecotypes (Creeper, Isfahan, Lari, Marand, Mashhad, Naked Neck, Sari, Shiraz, Yazd) and 2 commercial strains (White Leghorn, Arian)—identified immune-related genes such as interleukins (IL-3, IL-13, IL-5), interferons, and heat shock protein genes (HSP70, HSPA9, HSPH1, HSP90AB1, PLCB4), which were previously unrecognized as involved in environmental adaptive traits (122). African native chickens, due to their high genetic diversity and adaptability to harsh tropical environments, are potential candidates for breeding virus-resistant flocks amid climate change (123). Among free-range chickens and local native chickens from Ethiopia, Saudi Arabia, and Sri Lanka, two candidate selection regions are shared by all free-range chickens, and four unique candidate selective sweep regions are shared by local native chickens (only one contains the annotated genes *Tshr* and *Gtf2a1*). Moreover, genes on chromosome 23 include three immune-related genes: *Hpcal4*, *Trit1*, and *Mycl3* (124). The transcriptional responses of innate immune genes to NDV vary among chicken embryos at different developmental stages. The expression levels of *Ccl5*, *Mx1*, and TLR3 in Kuroiler chicken embryos are much higher than those in Tanzanian local ecotype chickens. The expression levels of *IRF-1* and *Stat* genes in the Ghs13 free-range chicken (Ghs13 free-range chickens are MHC-defined Ghs13 Leghorns raised with outdoor access) subline are higher, and the expression is subline-dependent—suggesting that the innate immune response may be genetically controlled by the MHC locus (125). As the core locus of avian immune inheritance, the MHC-B gene harbors SNP variations in two-thirds of its exons that directly affect the structure of the antigen-binding groove, thereby determining the antigen presentation efficiency against different pathogens (126, 127). In conclusion, indigenous chicken breeds possess superior genetic adaptability and stress tolerance. For instance, through long-term natural selection, representative indigenous populations from Africa and China have accumulated abundant functional variations in immune-related genes. The basal expression levels of their innate immune genes are significantly higher than those of commercially bred breeds, and their tolerance threshold to viral infections is significantly higher than that of commercial breeds. Artificial selection for high-yield traits in commercial broilers and laying hens has led to a reduction in the genetic diversity of immune-related genes (such as IL-3, IL-13, HSP70). Although production performance has improved, resistance to heat stress and viral infections has declined significantly, resulting in a negative correlation of high yield with immune deficiency. The genetic background determines individual differences in immune susceptibility among chicken populations, laying the intrinsic foundation for the occurrence and progression of subsequent viral infections.

### 3.2 Viral impact: network perturbation mechanisms of differential genes and pathways

#### 3.2.1 Common disturbance mechanism

Viruses, as key external disturbance factors in the immune cooperative network, achieve immune escape, replication, and spread by targeting core signaling nodes, regulating the expression of functional genes, and disrupting cooperative loops between modules. IBDV VP5 protein suppresses RLR-mediated innate immune signaling by disrupting nucleocytoplasmic transport and impairing IRF7/p65 nuclear translocation, thereby blocking MAVS-dependent IFN-*β* production (128). Avian immunosuppressive viruses (including IBDV, MDV, and ALV) all evade PRR-mediated IFN-I signaling pathways—including the IRF7/NF-κB signaling axis and the IFNAR-JAK–STAT pathway—and suppress the host’s innate antiviral immunity through “two-stage blocking,” defined here as blockade of PRR-mediated IFN induction and blockade of IFNAR-JAK–STAT signaling, which is the core common mechanism of avian viral immune escape (129). The JAK/STAT pathway is an important antiviral node, and targeted inhibition alleviates virus-induced host damage. A GWAS study on ALV-J-infected Chengkou mountain chicken hens identified significant SNP enrichment on chromosome 6, associated with genes (*Ptpn13*, *Ttf2*, *Tial1*, *Dlg2*, *Fbxl7*, *Cdh5*, *Cdh11*) involved in tumorigenesis and immunosuppression via JAK/STAT and cell adhesion molecule pathways. Candidate genes (*Ankh*, *Slc4a7*, *Slc5a1*) may regulate ALV-J infection through membrane transport and inflammatory modulation (130). ultimately leading to the failure of synergistic activation between innate and adaptive immunity. Imbalanced regulation of the NF-κB pathway is another key mechanism. Some avian viruses inhibit NF-κB activation to evade host immunity, while others trigger excessive NF-κB hyperactivation to aggravate inflammatory injury. For instance, IBDV infection causes abnormal activation of the TGF and MAPK pathways (131). ALV-J-induced differential expression of genes such as *Commd3*, *Ppp1cb*, *Vegfa*, *Gtf2h1*, *Notch2*, *Itpr1*, *Fgfr4*, *Gnas*, *Nectin1*, *Wnt2b*, *Ppp1cc*, and *Mrc2* (132) further exacerbates immunosuppression by regulating the ubiquitination and degradation of the NF-κB inhibitor. After ALV-J infection, antimicrobial peptide genes such as *Avbd1*, *Avbd6*, and *Cath1-3* are continuously differentially expressed (133), disrupting the “early pathogen clearance barrier” of innate immunity. Transcriptomic analysis revealed differential expression of these genes, but functional validation is still needed.

#### 3.2.2 Virus-specific disturbance characteristics

The perturbation of the immune network by different viruses shows a “module-specific preference” and is regulated by the genetic background (breed/strain) of chickens, forming a “virus-host” coevolutionary adaptation. Viral infection-induced immune network perturbation exhibits distinct characteristics determined by both pathogen types and host genetic backgrounds, with key features summarized in Table 2.

**Table 2:** Virus-specific immune disturbance characteristics in chickens.

Virus	Target pathway/module	Key genes	Breed specificity/functional notes	Cite
NDV	Adaptive immunity, antigen presentation	Blb2 (MHC-B), Dma	Polymorphisms strongly associated with IgY concentration and antibody titers; modulates adaptive immune perturbation magnitude	(231–233)
IBDV	Innate-adaptive immune transition	Cd86, Ctla4	Directly targets bursa of Fabricius (central hub for B cell maturation); inhibits T/B cell interactions	(234)
AIV (H5N1)	Innate immunity, PRR signaling	Mx1, Tlr3	Ri chicken (resistant strain): high expression enhances viral sensing and antiviral signal transduction	(140)
MDV	Adaptive immunity, T cell activation	Csta, Lag3, Tap1	FSIL hybrid (resistant line): maintains adaptive immune integrity via regulating T cell activation and antigen presentation	(235)
NDV vaccine	Adipose tissue immune response	Nr4a3	Hy-Line Grey chickens: altered expression during primary/secondary immune responses	(236)

This table summarizes the targeted immune pathways, representative key genes, breed-specific functional characteristics and relevant citations of typical avian viruses including NDV, IBDV, AIV and MDV, as well as NDV vaccine, highlighting distinct molecular and immunological responses across chicken breeds.

With the update and progress of technology, the core molecular nodes through which ALV-J regulates the immune-tumor pathway can be screened using single-cell sequencing combined with protein–protein interaction experiments. Chicken gene knockout models can be constructed using gene editing technology to verify the regulatory role of these nodes in multi-module collaboration, achieving a breakthrough in viral perturbation mechanisms from “listing differentially expressed genes” to “verifying network regulation”. By performing genome editing on immune-related genes of specific chicken breeds (Table 3), the key role of host factors in viral entry or replication can be revealed. Viral infection not only directly impairs immune organs and compromises immune function, but also further disrupts intestinal microecological balance and induces perturbations in the structure of intestinal microbial communities. Across all viruses, two common themes emerge: targeting of the JAK/STAT and NF-κB pathways, and breed-specific genetic polymorphisms that modulate susceptibility. However, each virus also exhibits unique pathogenic signatures: ALV-J links immune dysregulation to tumorigenesis, IBDV primarily targets the bursa of Fabricius, and AIV/MDV exploit distinct host resistance gene networks.

**Table 3:** Application of gene editing technology in chicken immunity.

Subject of study	Handling method	Key findings	Cite
Transgenic chicken model of GFP-labeled PGCs	Germ cell-mediated transgenesis technology	129I/130 N substitution in chicken ANP32A abolishes IAV polymerase support.	(237)
DF-1 Chicken Fibroblasts	Receptor gene editing	Edit tva, tvb, and chNHE1 to generate ALV A/B/J-resistant chicken cells; confirm no shared host receptors among ALV A, B and J subtypes.	(238)
White Leghorn chicken PGC transplantation model	W38 tryptophan deletion	Chickens lacking W38 are resistant to ALV-J infection.	(239)
Commercial laying hen breeds	chNHE1 targeted editing generates mutations, introduced into commercial chicken strains by transgenesis.	ALV-J-resistant genetically modified chickens have stably heritable mutations for large-scale breeding.	(240)
RAG1-deficient chicken embryos	Gene knockout	Impaired embryonic B cell development during proliferation is linked to abnormal Ig V(D)J rearrangement and BCR signaling.	(241)
Commercial Hy-line laying hens	Chicken ANP32A editing + ANP32B/ANP32E inhibition in cells.	It can prevent the spread of avian influenza virus among poultry.	(242)
DF-1 cell line	construction of PRMT5 gene-deficient cell lines	PRMT5 inhibits chIFN-β activation; knockout elevates IFN-β/related genes, repressing antiviral immunity via negative regulation of the IFN-β pathway.	(243)

An overview of published studies utilizing gene editing approaches to explore and improve chicken immune responses against viral pathogens. PGCs, primordial germ cells; IAV, avian influenza virus; ALV, avian leukosis virus; BCR, B cell receptor; chIFN-β, chicken interferon-β; PRMT5, protein arginine methyltransferase 5.

#### 3.2.3 miRNA-protein axis: core regulators of chicken immune network

MicroRNAs (miRNAs) serve as core epigenetic regulators in chicken immunity, bridging innate and adaptive immune responses through “one-to-many/many-to-one” targeting mechanisms. Post-pathogenic infection, miRNAs reprogram immune gene expression and coordinate with proteins to modulate immune networks, characterized by common pathway targeting as well as virus-specific and breed-specific regulatory preferences (134). Differentially expressed proteins (DEPs) form “miRNA-target gene-protein” regulatory cascades, with their expression and modification dually shaped by viral tropism and chicken breed genetics. Key examples include breed-specific antioxidant defense in Beijing-You chickens (135) and virus-induced disruption of redox balance or endoplasmic reticulum stress (136, 137). Despite these insights, current miRNA research is limited by cell-type specificity masking, unclear miRNA-DNA methylation crosstalk, and uncharacterized immune resistance markers (138). The key regulatory roles of miRNAs and differentially expressed proteins under pathogenic challenge are summarized in Table 4.

**Table 4:** Key regulatory miRNAs and differentially expressed proteins (DEPs) in chicken immune responses.

Molecule type		Pathogen/challenge	Target/pathway	Cite
miRNAs	miR-133a	–(Ross broiler)	Splenic inflammatory pathway	(244)
	miR-146b-5p	Salmonella spp.	NF-κB signaling pathway	(245)
	gga-miR-146a-5p/155/214b-3p	Newcastle Disease Virus (NDV)	Innate immune network	(246, 247)
	gga-miR-206/499	–	Cell-mediated/humoral immunity pathways	(248)
DEPs	ITGB3, MTOR	Marek’s Disease Virus (MDV)	Immune regulatory hub pathways	(249)
	GRP78, PERK	Infectious Bronchitis Virus (IBV)	ER stress/NF-κB pathway	(136, 250)
	PRDX4, GPX3	Reticuloendotheliosis Virus (REV)	Redox balance pathway	(137)
	CAT, SOD, PRDX	*Salmonella Typhimurium*	Antioxidant defense pathway	(135, 251)
	ALV-J-related proteins	Avian Leukosis Virus Subgroup J (ALV-J)	Ubiquitination/immune signaling pathways	(252)

This table summarizes the identified miRNAs and DEPs, corresponding pathogens, as well as their targeted immune, inflammatory, antioxidant and stress-related signaling pathways, with supporting literature citations.

Notably, miRNAs and DEPs do not act independently but form a coordinated regulatory network to modulate immune function. In a study of heat-stressed broilers, *miR-34a* expression correlated with increased IFN-*γ* production (139), suggesting a regulatory role, and its downstream signaling molecules (*Stat1*, *Irf7*) are differentially expressed at the protein level during AIV and NDV infections (140), and *Traf6*— a key mediator of the NF-κB pathway—cooperates with REV-upregulated PRDX4 to maintain immune-oxidative stress homeostasis; viral disruption of this balance contributes to immune dysfunction.

#### 3.2.4 Current limitations and future directions

First, most DEPs identified via omics screening lack *in vivo* functional validation using techniques such as CRISPR/Cas9 knockout, leading to unresolved discrepancies between *in vitro* and in vivo effects. Second, low-abundance key regulatory proteins in immune signaling pathways (e.g., cytokine receptors, transcription factors) are difficult to fully captured by conventional proteomic approaches, particularly standard shotgun proteomics, which often fails to detect these low-expression molecules due to interference from abundant housekeeping proteins. Notably, this conventional method also frequently misses critical post-translational modifications, such as phosphorylation and ubiquitination that are pivotal for immune signal transduction. To overcome these limitations, alternative techniques including targeted proteomics (e.g., parallel reaction monitoring, PRM) and single-cell proteomics offer higher sensitivity for quantifying low-abundance proteins and resolving PTM profiles at the cellular level, necessitating the integration of enrichment strategies such as co-immunoprecipitation coupled with mass spectrometry co-immunoprecipitation coupled with mass spectrometry (CoIP-MS). Third, research on PTMs (e.g., phosphorylation, ubiquitination, acetylation) remains fragmented, and the extent to which viruses coordinate with miRNAs to regulate immune networks via these modifications requires systematic investigation. Addressing these gaps will advance our understanding of host-pathogen interactions and inform the development of novel strategies to enhance chicken disease resistance. The miRNA-protein networks described here are not only perturbed by viruses but also modulated by intestinal microbiota metabolites, as discussed in the next section.

## 4 Regulation of chicken immunity by intestinal microbiota

### 4.1 Intestinal microbiota distribution and immune modulation in chickens

#### 4.1.1 Distribution characteristics of intestinal microbiota

The intestinal microbiota, a core component of chicken immune networks, forms a “microbiota-metabolites-immune signals” regulatory axis with the intestinal barrier (physical, chemical, microbial, immune layers) (141), governing immune homeostasis and disease resistance. The barrier’s functional integrity and microbiota dynamics synergize to establish a defense-regulation-immune response network. Mucosal damage (e.g., villus shortening, crypt deepening) and microbiota dysbiosis are mutually reinforcing; damage impairs barrier function, facilitating pathogen colonization, while dysbiosis exacerbates inflammation and mucosal injury. Intestinal epithelial junctional complexes (tight/adherens junctions, etc.) maintain barrier function; In chickens, key tight junction proteins include claudin-1, occludin, and ZO-1, whose downregulation under stress conditions correlates with increased paracellular permeability (142). Impaired tight junction protein expression causes intestinal leakage, facilitating pathogenic invasion (143), with complex integrity dependent on epithelial energy metabolism (144). Microbiota is highly plastic, susceptible to stressors (temperature, antibiotics, diet, pathogens) (145), microbial barrier imbalance induces redox disruption and chronic inflammation, impacting production efficiency. Defining microbiota imbalance remains challenging, requiring multi-dimensional indicators (structure, metabolites, immunity) rather than composition alone. Microbiota diversity/density vary across gastrointestinal segments, matching regional physiological functions. Some studies suggest that lower abundance of *Akkermansia muciniphila* and higher abundance of Lactobacillus are associated with improved feed efficiency in broilers (146), though causal relationships require validation. Microbiota imbalance involves reduced beneficial microbes (Lactobacillus, Clostridium) and overproliferation of potential pathogens (e.g., Proteobacteria), whose H₂S production damages epithelial cells and induces inflammation (147).

The distribution of chicken intestinal microbiota exhibits significant intestinal segment specificity, host dependence, and environmental responsiveness, forming a structured microbial ecosystem. Most existing studies are based on laboratory-simulated environments or specific ages and breeds, lacking long-term dynamic tracking data under different breeding models (large-scale breeding, organic breeding), regions, and climatic conditions. Additionally, most studies rely on 16S rRNA sequencing to analyze microbiota composition, with limited capacity to resolve species-level classification and differentiate metabolically active from dormant microbial populations. Future studies should integrate metagenomic shotgun sequencing to resolve species-level composition and metatranscriptomics to distinguish metabolically active from dormant microbiota. Most conclusions are based on overall microbiota analysis without targeted validation for individual genera/strains. Future research should deepen investigations into distribution and interaction mechanisms, construct a four-in-one analysis model of “genome-transcriptome-metabolome-microbiome,” and combine machine learning algorithms. For instance, random forest models have been used to predict feed efficiency based on cecal microbiota composition (148) to explore the association network between microbiota and host phenotypes (growth performance, egg production rate, disease resistance), achieving functional prediction and target screening. Intestinal microbiota dysbiosis can exacerbate immune impairment by regulating intestinal barrier function and inflammatory signaling pathways, while exerting synergistic effects with external environmental stressors. In addition to characterizing community composition, recent multi-omics studies have further revealed the functional potential and molecular mechanisms by which the chicken gut microbiota regulates host immunity and physiological functions (149, 150). For example, metagenome-assembled genome analysis of the Jianghan chicken gut revealed that Phocaeicola and Bacteroides are the most abundant bacterial genera, and the microbiota is dominated by metabolic genes, with the highest number of genes associated with carbohydrate metabolism (151); Butyrate produced in the chicken gut can regulate intestinal epithelial barrier function by upregulating the expression of tight junction proteins and inhibiting histone deacetylase activity (152). Metabolomics-related studies further corroborate these functional patterns, confirming that propionate regulates NF-κB signaling and immune homeostasis (153). Integrated multi-omics analyses based on 16S rRNA sequencing, metabolomics and transcriptomics further elucidated the cascade regulatory network: environmental stressors such as heat stress alter the abundance of gut microbiota-derived short-chain fatty acids, thereby modulating host intestinal immune responses and oxidative stress-related transcriptional profiles, and ultimately affecting poultry disease susceptibility (154). Based on the analysis of multi-omics technologies such as metagenomics and metabolomics, it has been found that the function of gut microbiota is no longer limited to the level of species composition. It can also actively regulate host immunity through metabolites and signaling pathways, providing theoretical support for systematically elucidating the interaction mechanism between gut microbiota and the host in chickens.

#### 4.1.2 Core regulation of dynamic changes in intestinal microbiota

The intestinal microbiota is regulated by the chicken’s developmental stage. The establishment of chicken intestinal microbiota begins in the embryonic stage: Emerging evidence suggests that the egg yolk harbors a low-biomass microbial community that may influence embryonic gut colonization (155), although the functional significance and potential contamination issues require further investigation. After hatching, the microbiota of newborn chicks mainly originates from hens (efficient donors of Bacteroidetes and Actinobacteria) and the environment (main sources of Lactobacillus, Erysipelotrichaceae, Helicobacteraceae, Ruminococcus) (156), and the complexity of the microbial community increases with age throughout the production lifecycle—particularly significant differences are observed during the rapid bone growth period (14–21 days of age) and rapid weight gain period (35–42 days of age). Analysis of the intestinal bacterial community diversity in Ross 308 broilers revealed that 90% of amplified sequence variants belong to the phyla Firmicutes and Proteobacteria, while over 90% of sequences in the fungal community are related to Ascomycota and others (157). The diversity and complexity of cecal microbiota in Arbor Acres broilers increase with age and gradually stabilize at 21 days of age. In one study of Arbor Acres broilers, Clostridium sensu stricto_1 dominated the cecal microbiota at day 1 (83.5% relative abundance), but this proportion decreased rapidly with age (158)—reflecting co-adaptation between the microbiota and host development. As age increases, microbial community succession becomes more complex, with similar dynamic changes observed in bacteria and fungi. Ross 308 broilers have a rich lung microbial community after hatching, whose composition changes with age: the lung index is highest at 3 days of age and then decreases, *α* diversity of the microbial community remains unchanged, while *β* diversity changes regularly with age. The relative abundance of Firmicutes and Lactobacillus increases, while that of Proteobacteria decreases—with dominant bacteria playing key roles, providing a basis for subsequent research on lung injury mechanisms (159).

Breed-specific genetic backgrounds lead to significant differences in microbiota structure among different chicken breeds. The intestinal microbiota of high-altitude Tibetan chickens is significantly different from that of low-altitude plum blossom chickens and Xuhai chickens: the genus *Bacilloplasma* and candidate genus *Bacilloplasma* are endemic to high and low altitudes, respectively. Luning chickens have the lowest *α* diversity but extremely high abundance of Firmicutes and Lactobacillus, and the microbiota structure of Wugu chickens is similar to that of Yaoshan chickens (23). Functional patterns differ significantly between broilers and laying hens: the carbohydrate metabolic pathway in the cecum of White Leghorn chickens is upregulated (conducive to egg production), while the genus *Paramyroides* in Silkie black-boned chicken affects metabolites, with upregulation of lipid metabolism and immune-related genes in the cecum (160). These differences essentially reflect selective shaping of microbiota structure by the host’s genetic background. In groups of brown-egg dwarf hens with higher feed efficiency, the abundance of Lactobacillus and *Akkermansia* in the cecum is significantly increased, while that of *Faecalibacterium* is decreased—and their microbiota is enriched in functions related to sugar metabolism and amino acid metabolism (161). Poultry breeds exhibit significant breed-specific differences in gut microbial composition and growth performance. Such variations are largely driven by host genetic factors, including inherent differences in intestinal mucin production, antimicrobial peptide synthesis, and immune receptor expression, which collectively regulate the colonization and structure of the gut microbiota (162). Stocking density is a critical environmental factor affecting animal health and microbial community structure. Increased stocking density induces chronic physiological stress, impairs intestinal mucosal integrity, and alters the secretion of immune-related molecules, thereby indirectly regulating gut microbiota assembly (163, 164). However, the specific molecular mechanisms underlying the interactions between stocking density, host genetics, and gut microbiota remain to be further elucidated in this study.

Breeding models also directly regulate microbiota structure and immunophenotype. The initial microbiota of newborn laying hens mainly originates from hens and the environment, but the influence of different sources on intestinal microbiota shaping remains unclear. Intestinal bacteria are mainly acquired through the hen’s reproductive tract (165). A comparison of Arbor Acres broilers raised in cages vs. on litter showed that litter-raised broilers have poorer early growth performance but better late-stage slaughter performance, with stronger peripheral and intestinal immune functions than caged broilers. Litter-raised broilers have higher ileal microbial community *α* diversity, as well as higher relative abundance of litter-associated bacteria and potential pathogenic bacteria—with these enriched microorganisms positively correlated with immune function, suggesting that moderate stimulation of microbial diversity may be key to enhancing immune function (166). Stocking density can affect bacterial diversity, abundance, and the distribution of major opportunistic pathogenic bacteria in the house, leading to reduced chicken growth rate and feed efficiency (167, 168). High stocking density increases airborne bacterial aerosols and fecal contamination, which may alter gut microbiota via the fecal-oral route and respiratory-gut axis. Bacterial aerosol concentrations (highest in summer, lowest in winter, with increased proportion of Gram-negative bacteria in autumn) (169) indirectly affect Intestinal microbiota balance and immune responses through the “respiratory-intestinal axis.” Dynamic changes in chicken intestinal microbiota are the result of coordinated regulation by multiple factors such as host genetics, nutritional supply, environmental factors, and microbial interactions. The maintenance of homeostasis is directly related to flock health and production performance. Most existing studies focus on the regulatory role of single factors (e.g., nutrition or genetics) and have insufficient understanding of the coevolutionary mechanisms among host genetics, microbiota, and the environment. Notably, current evidence regarding host genetics-microbiota coevolution remains largely correlational, and distinguishing causal relationships from mere associations requires well-designed longitudinal studies integrated with detailed phenotypic data. Future research should combine genome-wide association studies and multi-omics techniques to reveal the coevolutionary relationship between host genetics and microbiota, clarify the “host-microbiota-metabolites” regulatory loop under environmental stress, focus on analyzing the molecular interaction mechanisms between key functional bacteria (e.g., SFB, butyrate-producing bacteria) and host immune cells, further explore the types and functions of understudied bacteria, and clarify the relationship between intestinal microbiota diversity and poultry industry production efficiency.

#### 4.1.3 Intestinal microbiota metabolites mediate immune regulation

As the core signal carriers of “microbiota-host immunity” interactions, intestinal microbiota metabolites achieve precise regulation of the immune network by specifically binding to surface receptors of intestinal barrier cells and immune cells, initiating downstream cascade signaling pathways. These metabolites mainly include polysaccharides, bile acids, and short-chain fatty acids. Microbial-associated polysaccharides (e.g., lipopolysaccharide, peptidoglycan) and dietary polysaccharides fermented by gut bacteria both contribute to immune modulation, though via distinct PRRs (TLR4 for LPS, NOD2 for peptidoglycan). Collectively, these metabolites not only regulate the intestinal microenvironment but also act as key molecules for maintaining intestinal immune homeostasis and resisting pathogen invasion. Their regulatory mechanisms exhibit significant characteristics of “receptor specificity-pathway targeting-effect synergy”, with distinct species specificity in poultry (170, 171). Polysaccharides produced by intestinal microbiota metabolism play important roles in immune regulation and are core molecules regulating the function of intestinal gut-associated lymphoid tissue (172). Primary (chenodeoxycholic acid, cholic acid) and secondary (deoxycholic acid, lithocholic acid) bile acids are cholesterol metabolism products jointly regulated by the host and intestinal microorganisms, functioning as signaling molecules. Primary bile acids are metabolized and converted into secondary bile acids (deoxycholic acid, lithocholic acid) by the Intestinal microbiota, forming a “host-microbiota” co-regulated bile acid pool. An interaction network between the intestinal microbiota and innate immunity is constructed through two major receptor families: GPBAR1 (TGR5) and farnesoid X receptor (173). Chickens possess primary bile acid patterns analogous to mammals, with chenodeoxycholic acid and cholic acid as the predominant primary bile acids. However, Chickens share similar primary bile acid composition with mammals, consisting mainly of cholic acid and chenodeoxycholic acid while their secondary bile acid profiles differ significantly, which is primarily determined by differences in gut microbiota composition (174). Gut microbiota mediates the deconjugation, dehydrogenation and dehydroxylation of primary bile acids to generate secondary bile acids, thereby shaping the unique bile acid metabolic signature in poultry, which further affects intestinal immune homeostasis and microbial-host crosstalk.

SCFAs—including acetate, propionate, and butyrate—are the main products of Intestinal microbiota fermenting dietary fiber. Their immune regulatory mechanisms are characterized by multiple targets, pathways, and effects, with significant differentiation in the regulatory functions of butyrate, propionate, and acetate. SCFAs inhibit excessive inflammation through two core pathways: Butyrate acid inhibits the activity of HDAC (histone deacetylase), induces histone hyperacetylation, and thereby silences and suppresses the transcription and expression of pro-inflammatory target genes such as IL6 and TNF-*α* downstream of the NF-κB signaling pathway, ultimately alleviating intestinal inflammation (175–177) or activating the AMPK/mTOR signaling pathway via GPR41/GPR43 receptors, inhibiting macrophage polarization to the M1 type and promoting M2 type polarization (anti-inflammatory phenotype) (178, 179). Acetate and propionate can promote Treg cell differentiation in the spleen and intestinal lamina propria (upregulating *Foxp3* expression) through GPR43-mediated signaling, enhancing their immunosuppressive function while inhibiting Th17 cell proliferation to maintain Th17/Treg balance (180). While well-established in mice, the role of GPR43 in chicken Treg differentiation requires experimental confirmation using chicken-specific reagents. Butyrate can directly promote DC secretion of IL-23 and regulate the cytokine secretion profile of *γ*δ T cells (enhancing IL-17 and IFN-γ production)—a regulatory mode particularly important in chicken intestinal immunity (γδ T cells account for over 50% of total intestinal T cells). Meanwhile, as the preferred energy source for intestinal epithelial cells, butyrate can enhance the integrity of the intestinal physical barrier by increasing intracellular ATP levels, promoting the synthesis and assembly of tight junction proteins (181), inhibiting histone deacetylase, and upregulating the expression of anti-inflammatory genes (e.g., IL-10, TGF-*β*) in intestinal epithelial cells—reducing immune disorders caused by intestinal leakage. In addition, acetyl-CoA carboxylase, as a key downstream molecule of the SCFA regulatory network, has its activity inhibited by AMPK-mediated phosphorylation (182), thereby affecting long-chain fatty acid synthesis. Meanwhile, long-chain fatty acid metabolites (e.g., arachidonic acid) can regulate immune cell inflammatory responses, forming a cross-pathway regulatory network—though the integrity of this mechanism in chickens remains to be verified. Intestinal microbiota metabolites do not function independently but exhibit coordinated regulation among metabolites. Currently, whether there is functional redundancy in the regulation of the same immune pathway (e.g., NF-κB) by different metabolites (e.g., SCFAs, polysaccharides), as well as their synergistic or antagonistic relationships, remains unclear. The immune regulatory effects of low-abundance metabolites (e.g., tryptophan derivatives such as indoleacetic acid, polyamines) have not been fully analyzed due to limitations in detection techniques. Additionally, there is a lack of precise analysis of metabolite-mediated regulatory pathways—for example, differences in specific targets of different SCFA components (acetate, propionate, butyrate) and the dose-effect relationship of secondary bile acids in regulating immune metabolism have not been clarified. Future research can verify the specific receptors and downstream signaling pathways of metabolites (e.g., secondary bile acids, indole derivatives) through *in vitro* cell co-culture, gene editing, and other technologies, clarifying the target sites and dose-effect relationships of different regulatory factors. Whether different SCFAs (acetate, propionate, butyrate) act redundantly or synergistically on the same immune pathway remains unclear, as most studies test individual metabolites rather than physiological mixtures. Targeted metabolomics using LC–MS/MS with isotope-labeled internal standards can quantify low-abundance metabolites such as indole derivatives and polyamines, which are emerging as key regulators of intestinal immunity.

### 4.2 The impact of microbiota transplantation on immune network remodeling

**Figure 3:**
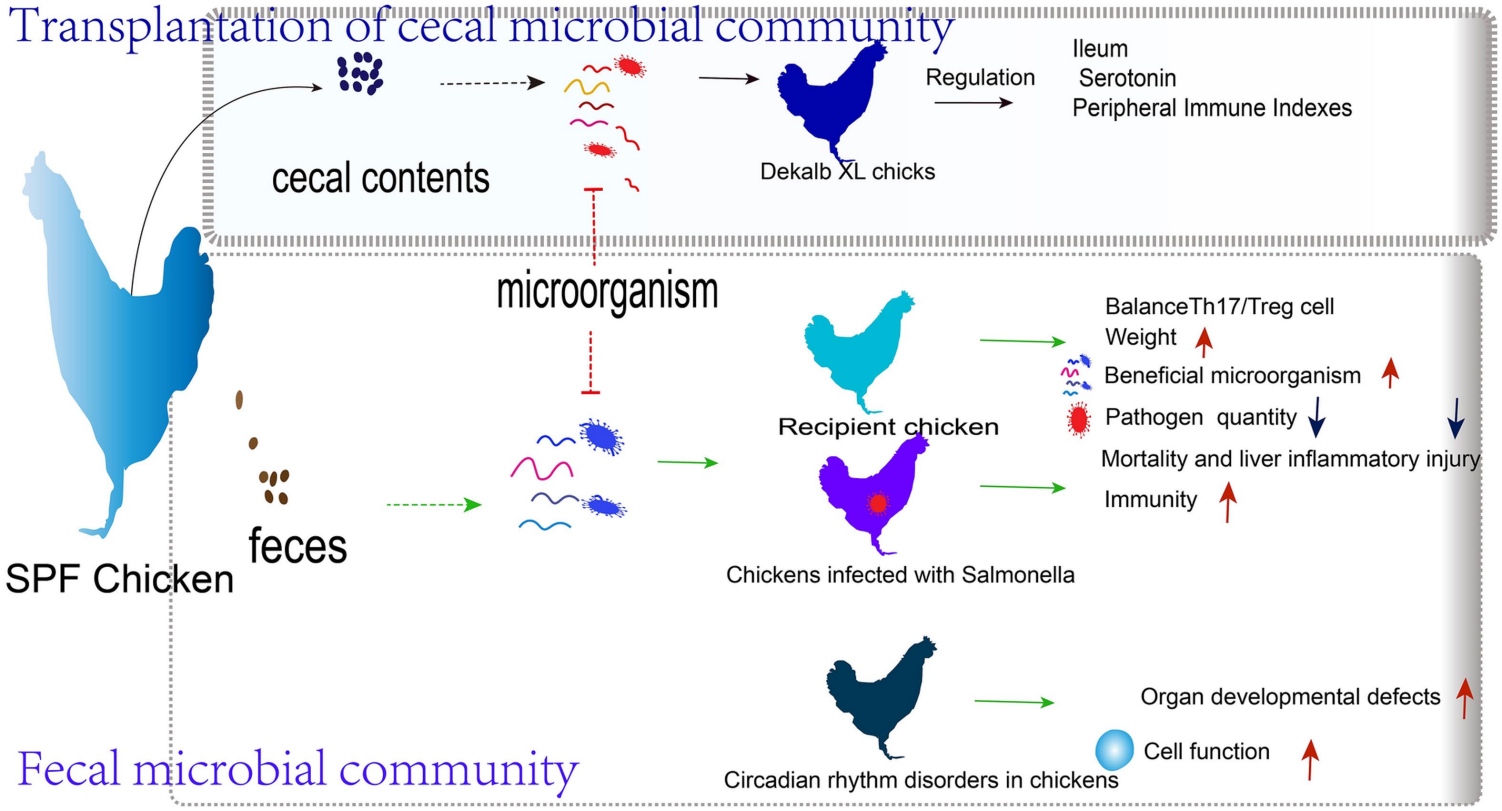
Schematic of cecal and fecal microbial transplantation and their immunomodulatory effects in chickens. Cecal microbial transplantation from SPF chickens regulates ileal serotonin and peripheral immune indices in Dekalb XL chicks. Fecal microbial transplantation enhances immunity, reduces pathogen load and liver inflammation in Salmonella-infected chickens, restores Th17/Treg balance in healthy birds, and mitigates organ defects in chickens with circadian rhythm disorders.

Microbiota transplantation typically activates the immune response system and optimizes chicken immune function by reshaping intestinal microbiota balance, exerting an indirect impact on the prevention and treatment of various diseases. As a core technology for precise intestinal microbiota intervention, it achieves functional remodeling of the chicken immune network by reconstructing microbiota structure and optimizing metabolite profiles. Its regulatory mechanism exhibits dual characteristics of “intervention target specificity” and “immune function targeting”, mainly including fecal microbiota transplantation (FMT) and cecal microbiota transplantation (CMT). FMT uses fecal material from donors, whereas CMT adopts cecal contents collected from donor animals. In a study using Five-black chicken with experimentally induced circadian rhythm disorders, FMT restored organ development and cellular function (183), which also provides new insights for poultry immunization research. FMT may promote weight gain, enhance pathogen tolerance, and increase the thickness of the serosa and muscle layers in broilers to improve intestinal morphology (184). It influences host health by increasing beneficial microbial communities, competitively excluding pathogens, reducing the production of growth-inhibiting metabolites by the microbiota, and improving energy metabolism (185). Transplanting the fecal microbiota of adult SPF chickens into chicks infected with *Salmonella Enteritidis* revealed that FMT could reduce the mortality rate and liver inflammatory damage of infected chicks, enhance immunity, and inhibit pathogen colonization (186). The regulatory effects of FMT on early intestinal immunity in chicks include increasing immune organ size, elevating SCFA levels, and enhancing intestinal barrier function (187, 188). The beneficial effects of FMT are donor-dependent; transplantation from healthy, disease-resistant donors is critical for success. CMT experiments demonstrated that early CMT from different donors could regulate ileal morphology, serotonin activity, and peripheral immune indicators in Dekalb XL chicks, thereby affecting their growth and health status (189). Although both techniques involve microbiota transplantation, FMT focuses on regulating jejunal Lactobacillus abundance and Th17/Treg balance to improve growth performance, while CMT mainly influences ileal morphology, serotonin activity, and peripheral immune indicators in recipient laying hens. The differences in immune regulatory effects between FMT and CMT essentially stem from the distinct sources of transplanted microbiota and donor characteristics (Figure 3). Fecal samples have limitations as substitutes for chicken intestinal microbiota studies, requiring caution in metagenomic analyses. The gut microbiome, characterized by diversity and adaptability, necessitates integration of host-microbiota interaction mechanisms, bioinformatics, and ecological methods. Microbiota transplantation—e.g., transplanting disease-resistant Beijing-You chicken intestinal microbiota—enhances susceptible flocks’ resistance to *Salmonella* and *Escherichia coli*, and optimizing FMT/CMT protocols with early colonization patterns promotes chick intestinal development and immune maturation. Three key challenges persist: lack of core markers for “highly efficient immune regulatory” donors (specific bacterial genus combinations, metabolite profiles) leading to unstable transplantation effects; unclear colonization/adaptation mechanisms of transplanted microbiota against native microbiota competition and host intestinal barrier; and large-scale operation (preparation preservation, administration) and cost issues restricting industrial application. Future research should identify core functional flora and markers via multi-omics (metagenomics, metabolomics, immunomics), optimize protocols, and advance translation from laboratory to large-scale breeding.

## 5 The influence of exogenous additives and the environment on chicken immunity

### 5.1 Additives

Although antibiotics can improve animal gastrointestinal function and enhance production efficiency, excessive use leads to drug resistance and residues, posing a threat to consumer health. Many countries and regions have enacted laws banning their use in animal feed, making the search for feasible substitutes a key research focus in the post-antibiotic era for poultry (190, 191). Feeding chickens with diets supplemented with probiotics or phytochemical growth promoters significantly alters multiple ileal intestinal metabolites, which are associated with host immunity, intestinal integrity, and muscle growth (192, 193). Glucose oxidase, is considered a safe and environmentally friendly feed additive based on current toxicological data (194). Although different types of additives (plant-based, fat-derived, enzyme preparations, insect proteins, amino acids, polysaccharides, etc.) act on distinct target sites, they modulate immune function by reshaping intestinal microbiota structure, optimizing metabolite profiles, and enhancing intestinal barrier function (195, 196). These additives exert synergistic effects through shared downstream pathways, including SCFA production, TLR modulation, and NF-κB inhibition, rather than merely exhibiting an undefined synergistic regulation of immunity. To systematically clarify the specific effects of different alternative additives on broiler immune function and growth performance, the following Table 5 summarizes the core research findings of representative additives.

**Table 5:** Summary of feed additives and their immune-related effects in broilers.

Category	Additive	Test animals	Core effects (extracted from original text)	Cite
Plant-derived	Plant extracts, Plant polysaccharides	Broilers	Enhance immunity and antioxidant capacity; promote immune organ development and growth	(253)
	*Lagenaria siceraria* (Molina) Standl	Sanhuang broilers	Induce Treg/Th2 immunity; enhance cytokine and antibody production; regulate gut microbiota and SCFAs; improve infection/inflammation resistance	(254)
	Prebiotic mixture (mannanoligosaccharides + garlic water extract), Mannanoligosaccharides alone	Ross 308 broiler embryos (injected on day 12 of incubation)	Regulate intestinal/liver cytokines; upregulate splenic AvBD1 and IL1-β	(255)
	Purple corn anthocyanin extract	Chishui black-bone chickens	Elevate plasma immunoglobulins and complements; increase bursa weight; alter immune-related gene expression	(256)
	Pulicaria jaubertii powder	Ross 308 broilers	Improve growth; modulate gut microbiota and intestinal morphology; boost mucosal immunity and IBD antibodies	(257)
	Echinacea extract + Astragalus polysaccharides	Broilers	Regulate amino acid and pentose metabolism; increase SCFAs; enhance mucosal barrier and immunity; counteract immunosuppression	(258)
	Puerarin	Pathogen-free White Leghorn broilers	Alter serum metabolites; regulate amino acid/lipid metabolism post-Salmonella enteritis	(259)
Fat-derived	Oxidized fats	Broilers	Upregulate mRNA expression level of IL-22 in the ileum	(260)
	Lauric acid	1-day-old broilers	Improve growth and immunity; regulate antibodies, cytokines, lipid metabolism and gut microbiota	(194)
Enzymes	Glucose oxidase (used alone or combined with *Bacillus amyloliquefaciens* SC06)	Broilers	Safe and eco-friendly; enhance antioxidant and immune function; improve cecal microbial environment	(261–263)
	Muramidase	Ross 308 broilers	Increase duodenal goblet cells and IELs; reduce CD3^+^ T cells; activate Nod2 via peptidoglycan hydrolysis; improve intestinal health	(264)
Insect protein	Black soldier fly larvae	Yellow-feathered broilers (0–6 weeks old)	Promote CD8^+^ lymphocyte proliferation; accelerate early viral clearance	(265)
Other functional additives	Glutamine	Broilers	Improve intestinal mucosal structure/function under stress (e.g., necrotic enteritis)	(266)

This table categorizes common functional additives into plant-derived, fat-derived, enzymes, insect protein and other types, and summarizes their tested chicken breeds, as well as core functions including immunity regulation, antioxidant capacity improvement, gut microbiota modulation, intestinal barrier maintenance and metabolic homeostasis, with corresponding literature citations provided.

**Figure 4:**
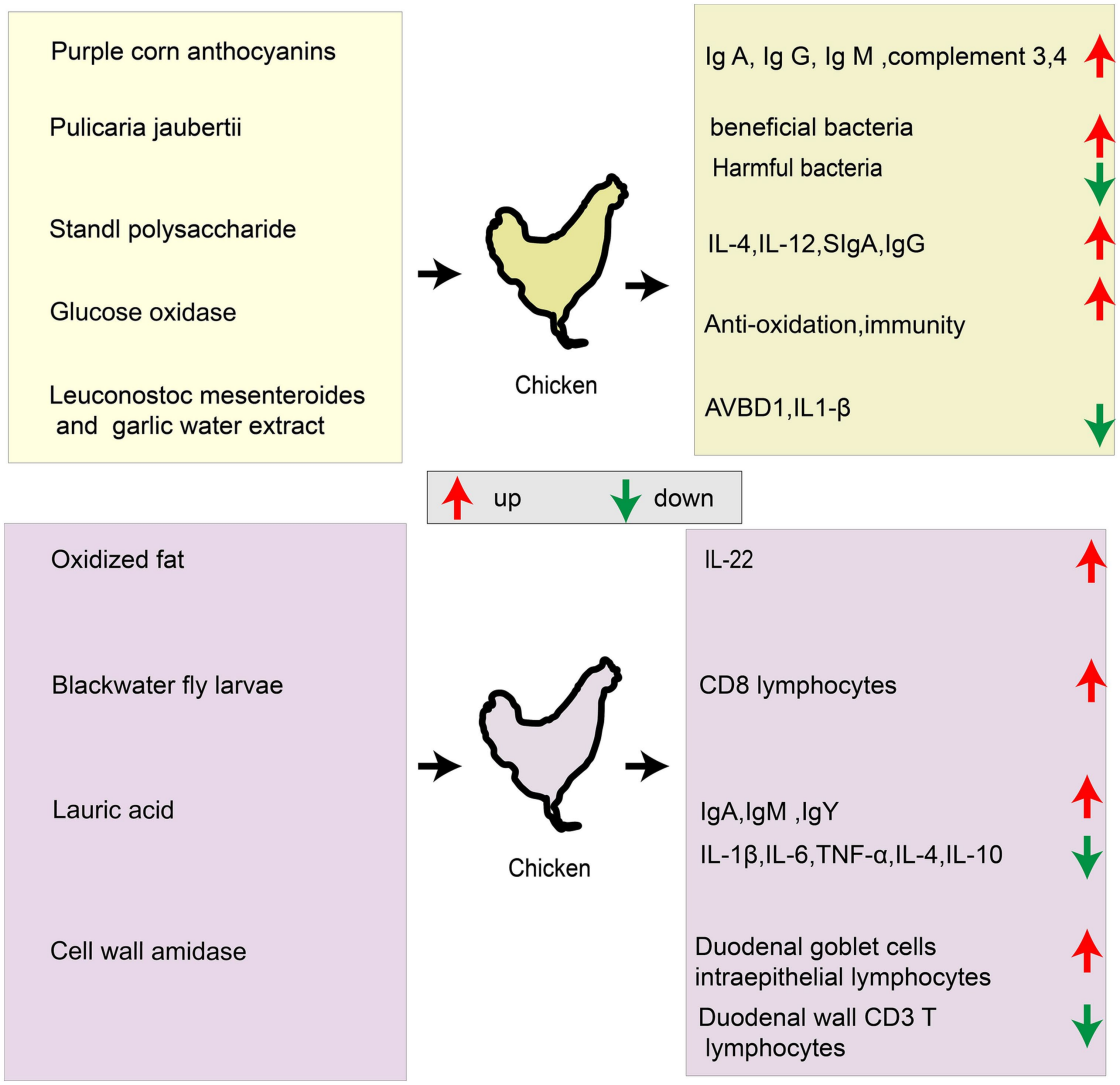
Schematic of immunomodulatory effects of functional and dietary interventions in chickens. Beneficial interventions (e.g., plant extracts, probiotics) enhance antibody levels, beneficial microbiota, and antioxidant capacity while reducing inflammation. Alternative interventions (e.g., insect meal, fatty acids) modulate cytokine profiles and intestinal immune cell populations, with red/green arrows indicating upregulation/downregulation.

Notably, different plant-derived additives exert targeted immunomodulatory effects: anthocyanin extract elevates serum immunoglobulins and promotes bursa development, *Pulicaria jaubertii* powder enhances mucosal immunity and specific antibody production, and Echinacea extract plus Astragalus polysaccharides boost SCFA synthesis to reinforce the intestinal barrier. Most current studies only confirm that additives can alter microbiota structure or immune indicator changes, but fail to clarify the specific contributions of core functional microbiota or the mediating role of key metabolites. The specific binding mechanisms of metabolites to immune cell receptors (e.g., GPBAR1, FXR, FFAR2/3) after additive-induced microbiota reshaping are insufficiently studied, especially lacking evidence of chicken-specific molecular interactions (Figure 4). Meanwhile, the action pathways of different additives overlap, but the molecular basis of their synergistic or antagonistic effects has not been analyzed—resulting in fragmented mechanism research and difficulty in forming a unified regulatory network. In the future, research focus can shift from describing microbiota structure to precisely locating functional microbiota. Through causal chain verification (screening, colonization, knockout), the core microbiota and metabolite targets mediating additive-induced immune regulation can be clearly identified.

### 5.2 Heat stress

Heat stress, as a critical external stressor, significantly disrupts the behavior, physiology, and immune regulatory mechanisms of poultry. Behaviorally, it is characterized by panting; physiologically, it leads to reduced feed intake and weight loss, ultimately potentially causing decreased feed conversion rate, growth retardation, and impaired meat quality (117, 197). Intestinal heat stress damages the intestinal tract, reducing the ratio of intestinal villus height to crypt depth and decreasing the number of proliferating cells—thereby disrupting the intestinal barrier and triggering inflammatory responses (198). Heat stress resulted in lower weight gain, increased abdominal fat rate, decreased thigh muscle rate, high water loss in the pectoralis major muscle, reduced antioxidant capacity, downregulated mRNA expression of intestinal barrier-related proteins, and increased relative abundance of Anoxybacter in the cecum of Huaixiang broilers. Nineteen differential metabolites were identified, mainly enriched in 10 signaling pathways (199). Regulating genetics, management, and nutrition is a potentially effective approach to alleviate heat stress-induced damage (200, 201). A high ambient temperature of (31 °C) 3 constituted mild heat stress significantly reduced the abundance of Christensenellaceae and Lachnospiraceae in the cecum of Arbor Acres broilers, while increasing the proportion of Peptococcaceae. These microbiota changes directly affect the production of SCFAs, with acetic acid content significantly increasing in the high-temperature group (202). The abundance of Ruminococcus and Clostridium was significantly elevated. Elevated Ruminococcus abundance under heat stress is associated with increased acetic acid production, which may promote regulatory T cell differentiation via GPR43 signaling. The impact of heat stress on immune cells is also reflected in changes in immune cytokines and immune organs. Twenty-two-day-old white-feathered broilers were exposed to moderate heat stress 34.5 ± 0.5 °C for 14 days. The number of Bu1^+^ B cells and CD3^+^ T cells in the spleen decreased, as did the number of related precursor cells in the thymus and bursa of Fabricius. Additionally, the morphology of the thymic cortex and bursa of Fabricius follicles was damaged. Following bovine serum albumin antigen immunization, the titers of anti-BSA IgY, IgM, and IgA antibodies were lower than those in the thermoneutral group (203). Heat stress increased the mRNA abundance of pro-inflammatory cytokines such as IL-1β, IL-4, IL-6, and TNF-*α* in the spleen of broilers. The growth index of lymphoid organs in yellow-feathered broilers exposed to severe heat stress 37 ± 2 °C decreased (204). This indicates that heat stress impairs the development and maturation of T and B cells, leading to various immune abnormalities in broilers. Reduced bursa weight can affect B cell development and maturation, decreasing antibody production capacity; reduced thymus weight will impair T cell differentiation and selection, weakening cellular immune function. From a molecular mechanism perspective, this may involve a series of signaling pathway alterations induced by heat stress—such as affecting the expression of cell cycle regulatory genes and apoptosis-related genes—thereby leading to reduced immune organ cell numbers and functional impairment.

**Figure 5:**
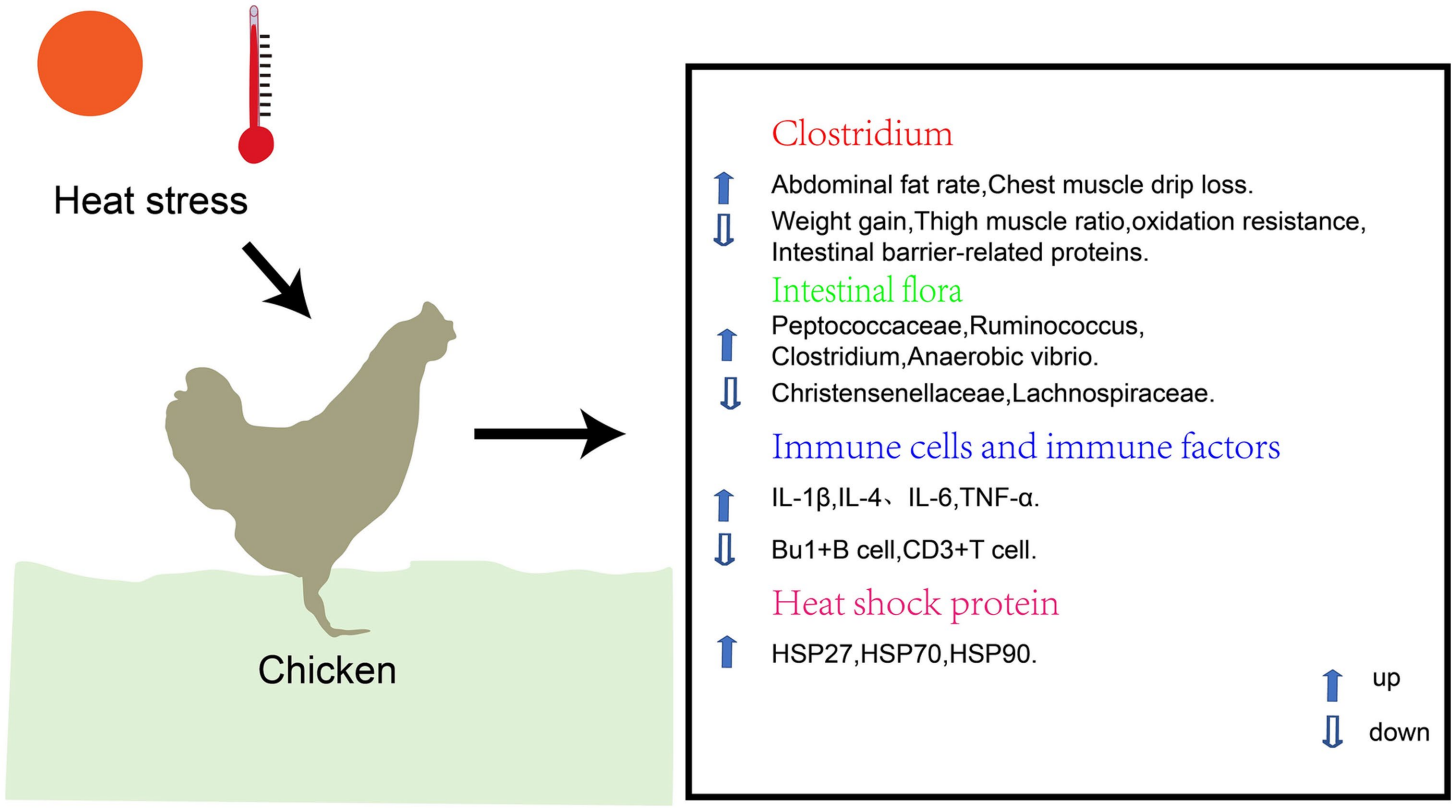
Impact of heat stress on chicken physiology, gut microbiota, and immunity. Heat stress impairs growth performance and meat quality, shifts gut microbiota composition toward pro-inflammatory taxa, elevates pro-inflammatory cytokines, reduces adaptive immune cell counts, and induces heat shock proteins (HSP27, HSP70, HSP90) in chickens.

Under heat stress conditions, the levels of *Hsp27* and *Hsp70* expression is significantly upregulated, while dietary astaxanthin supplementation downregulates their expression (205). *HSP60* critically regulates the production of endogenous IL-1β in activated microglia by stimulating the *Nlrp3* inflammasome pathway (206). Heat shock proteins can induce immune cells, monocytes, macrophages, and dendritic cells to release pro-inflammatory cytokines (207). Heat stress exposure increased the protein levels of IL-1β and TNF-*α* in the chicken spleen, while decreasing the levels of IL-2 and IFN-*γ* (208). Increased intracellular HSP expression can enhance cell tolerance to inflammatory cytokines produced under heat stress conditions. CD91 is a common receptor for HSPs (gp96, *Hsp90*, *Hsp70*) and calreticulin, and *Hsp70* also regulates adaptive immunity by presenting stress cell peptide fragments to cytotoxic T cells (209). After 15 days of high-temperature exposure, the mRNA expression of *Hsp27*, *Hsp70*, and *Hsp90* in the bursa of Fabricius and spleen of 42-day-old black-bone chickens was upregulated (210). While HSPs can act as danger signals to activate innate immunity, chronic heat stress-induced overexpression of HSP70 in lymphoid organs correlates with apoptosis and reduced lymphocyte counts, suggesting a context-dependent role. Transfecting *Hspa8* overexpression plasmids, interference fragments, and corresponding controls into chicken macrophage cell line (HD11) cells revealed that significantly upregulated *Hspa8* expression in HD11 cells inhibited LPS-induced IL-1β and NF-κB expression (211). This indicates that *Hspa8* promotes HD11 cell proliferation and inhibits apoptosis, exerting a pro-inflammatory effect. In addition to HSP-mediated immune regulatory disorders, heat stress often acts synergistically with other external factors. Researchers identified 48 potential heat stress-related genes from transcriptome sequencing data. Subsequent experiments using bone marrow dendritic cells from White Leghorn chickens combined with heat stress and LPS showed that the combined effect of heat stress and LPS reduced the Expression of genes such as *Crhr1*, triggering cellular pro-inflammatory responses. LPS exacerbates heat stress-induced immunosuppression and prolongs the time required for cells to adapt to stress (212). Meanwhile, heat stress is often accompanied by oxidative stress. When the body produces reactive oxygen species exceeding its own antioxidant capacity, oxidative stress occurs. In the pectoralis major muscle of heat-stressed broilers, heat stress aggravates lipid peroxidation and protein oxidation, and alters antioxidant enzyme activity (213). Heat stress is one of the most significant environmental stressors in poultry farming, especially in large-scale high-density breeding models. It significantly affects production performance by disrupting chicken physiological metabolism, immune homeostasis, and intestinal microecological balance (Figure 5). Current research has clearly identified the core dimensions of heat stress impacts on chicken production performance and immune regulation, forming a preliminary research system encompassing phenotypic changes—molecular mechanisms—intervention measures. However, significant shortcomings remain in “mechanism depth, breed specificity, and intervention precision”. How the HPA axis and oxidative stress pathways activated by heat stress precisely regulate the transcriptional expression of immune molecules (e.g., antimicrobial peptides, cytokines), as well as key regulatory molecules (e.g., interactions between transcription factor NF-κB and stress signals), remain unclear. Research on breed and stage specificity is insufficient, and universal conclusions are questionable. Future research should focus on three core aspects—mechanism deepening, precise intervention, and breed adaptation—to construct a complete regulatory network linking stress signals—metabolism—immunity—production performance. Environmental stress can amplify the adverse effects of genetic susceptibility, viral invasion, and microbiota dysbiosis. These four factors are interconnected, share multiple immune and inflammatory regulatory pathways, and collectively contribute to the regulation of immune homeostasis in poultry. In addition to heat stress, litter type, microbial exposure, rearing mode and feed environment are also crucial external environmental factors for poultry, which can likewise affect gut microbial community structure, intestinal barrier function and host immune homeostasis.

### 5.3 Environmental pollutants

With the development of industrialization and agricultural modernization, the environment for raising domestic chickens is facing the threat of various pollutants. Soil pollution from growing feed and pollution during the feed processing have led to an increase in the content of heavy metals in the feed. Environmental pollutants can have a significant impact on the immune system of chickens. For instance, arsenic trioxide exposure causes mitochondrial DNA in chicken liver cells to leak into the cytoplasm, thereby activating the cGAS-STING pathway and IFN-I response. Meanwhile, STING promotes the activation of the traditional NF-κB signaling pathway, triggering the expression of pro-inflammatory cytokine genes. Clearing mtDNA with ethidium bromide and inhibiting the cGAS-STING pathway with H-151 can effectively reverse the innate immune and inflammatory responses induced by arsenic trioxide (214). In chicken hepatoma cells, *miR-29a-3p* responds to selenium and inhibits the RhoA/ROCK signaling pathway by focusing on the *Col4a2* gene (215). Studies have shown that the inflammatory conditions induced by various harmful gas molecules (such as NH_3_, H_2_S, etc.) are closely related to the activated NF-κB pathway. After broilers were exposed to NH_3_, the mRNA and protein expressions of inflammatory factors such as NF-κB, cyclooxygenase-2, PTGES and inducible nitric oxide synthase increased. The *miR-6615*/*Smad7* axis was involved in the inflammatory injury induced by NH3 through the NF-KB pathway in the kidneys (216). NH_3_ stimulates changes in the cytokines secreted by Th2 and Th17, thereby exacerbating the imbalance of Treg and leading to tracheal inflammation (217). H_2_S exposure triggers tracheal inflammatory injury in chickens through *Fos*/*IL8* signaling mediated by oxidative stress (218). H_2_S triggers immune damage by activating the TLR-7/MyD88/NF-κB pathway and *Nlrp3* inflammasome in the thymus of broilers (267), and H₂S exposure induces apoptosis and necroptosis through *lncRNA3037*/*miR-15a*/BCL2-A20 signaling in the trachea of broilers (219). Excessive NH_3_ treatment of chicken thymus led to immunosuppression, metabolic disorders and apoptosis (220). NH₃ disrupts the balance between regulatory T and Th1 cells, manifested as decreased Treg-mediated immunosuppression and increased secretion of Th1-type proinflammatory cytokines (216). Antibiotic resistance genes (ARGs) are emerging environmental contaminants. While ARGs do not directly attack immune cells, their horizontal transfer among gut bacteria can alter microbiota composition and indirectly affect immune homeostasis. Rather, they do not directly act on immune cells themselves, the proliferation and spread of bacteria carrying ARGs in the chicken intestines can change the intestinal microbiota (221). The relative abundance of ARGs in the cecum, feathers and chicken coop samples of laying hens is high, and the ARGs in the cecum mainly originate from the chicken coop. The main bacteria affecting the changes of ARGs in the cecum, such as *Klebsiella pneumoniae*, were identified, and these bacteria were transmitted from the chicken coop to the cecum (222). In conclusion, environmental pollutants disrupt the immune metabolism and intestinal health of domestic chickens through multiple mechanisms, thereby affecting their growth performance and disease resistance. Future research needs to further explore the mechanism of action of pollutants and develop corresponding mitigation strategies to reduce their negative impact on the poultry farming industry. As a promising intervention strategy, nanoadjuvants are capable of mitigating the negative impacts of environmental pollutants on immune function and intestinal health. Featuring unique nanoscale delivery systems, such as size-controllable polymeric nanoparticles and pathogen-mimicking nanostructures, novel nanoadjuvants provide an innovative approach to enhancing the magnitude and durability of immune responses in chicken vaccines, mainly by improving antigen presentation efficiency and activating TLR/NLR signaling pathways. Nanoadjuvants, including types such as nanoparticles and nanoemulsions, play a crucial role in the vaccine immunization process (223). Not only did it enhance the immunogenicity and stability of the vaccine, but it also significantly strengthened the immune response through targeted delivery and activation of immune signaling pathways. These characteristics provide new ideas and methods for developing more efficient and stable poultry vaccines.

## 6 Conclusion

Multiple factors including viruses, intestinal microbiota dysbiosis, heat stress and environmental pollutants exert synergistic impacts on chicken immunity. Viral infection combined with heat stress damages the intestinal barrier and disrupts microbiota structure, which further aggravates the toxic effects of environmental pollutants and weakens host detoxification capacity. These interactive relationships construct an integrated immunity-microbiome-environment axis that dominates immune homeostasis in domestic chickens. Current strategies involving genetically engineered vaccines, nano-immune enhancers, probiotics, intestinal microbiota transplantation, and optimized feed formulas have shown promising effects on regulating intestinal flora, enhancing immunity and antioxidant status.

## 7 Future perspectives

Despite these advances, three key challenges remain: the molecular mechanisms underlying multi-factor synergistic damage are still unclear; targeted interventions for combined stress are insufficient; and the practical stability of emerging technologies in large-scale breeding requires further verification. Moreover, matching stage-specific nutritional supply with immune improvement and economic cost is critical for industrial application. Specifically, it is necessary to adjust the proportion of protein, energy and immune-enhancing nutrients according to the different physiological needs of broiler chicks, growing broilers and laying hens.

Future research should prioritize clarifying the cross-talk mechanisms of multiple stressors, developing precise immune regulation techniques, and validating the applicability of vaccines, adjuvants and microecological preparations under commercial production conditions. Multidisciplinary integration of immunology, microbiology, nutrition and precision livestock farming will help formulate practical strategies to sustainably improve flock health and resilience.
